# *Phytophthora infestans* RXLR effectors act in concert at diverse subcellular locations to enhance host colonization

**DOI:** 10.1093/jxb/ery360

**Published:** 2018-10-16

**Authors:** Shumei Wang, Hazel McLellan, Tatyana Bukharova, Qin He, Fraser Murphy, Jiayang Shi, Shaohui Sun, Pauline van Weymers, Yajuan Ren, Gaetan Thilliez, Haixia Wang, Xinwei Chen, Stefan Engelhardt, Vivianne Vleeshouwers, Eleanor M Gilroy, Stephen C Whisson, Ingo Hein, Xiaodan Wang, Zhendong Tian, Paul R J Birch, Petra C Boevink

**Affiliations:** 1 Division of Plant Science, University of Dundee at James Hutton Institute, Invergowrie, Dundee, UK; 2 Cell and Molecular Sciences, James Hutton Institute, Invergowrie, Dundee, UK; 3 Key Laboratory of Potato Biology and Biotechnology, Ministry of Agriculture, Huazhong Agricultural University, Wuhan, China; 4 Heilongjiang Bayi Agricultural University, Daqing, China; 5 Virus-free Seedling Research Institute of Heilongjiang Academy of Agricultural Sciences, Harbin, China; 6 School of Life Sciences Weihenstephan, Technische Universität München, Freising, Germany; 7 Wageningen UR Plant Breeding, Wageningen University, Wageningen, The Netherlands

**Keywords:** Avirulence, biotrophy, effector-triggered susceptibility, pathogenicity, Phytophthora, RXLR effector, virulence

## Abstract

Oomycetes such as the potato blight pathogen *Phytophthora infestans* deliver RXLR effectors into plant cells to manipulate host processes and promote disease. Knowledge of where they localize inside host cells is important in understanding their function. Fifty-two *P. infestans* RXLR effectors (PiRXLRs) up-regulated during early stages of infection were expressed as fluorescent protein (FP) fusions inside cells of the model host *Nicotiana benthamiana*. FP–PiRXLR fusions were predominantly nucleo-cytoplasmic, nuclear, or plasma membrane-associated. Some also localized to the endoplasmic reticulum, mitochondria, peroxisomes, or microtubules, suggesting diverse sites of subcellular activity. Seven of the 25 PiRXLRs examined during infection accumulated at sites of haustorium penetration, probably due to co-localization with host target processes; Pi16663 (Avr1), for example, localized to Sec5-associated mobile bodies which showed perihaustorial accumulation. Forty-five FP–RXLR fusions enhanced pathogen leaf colonization when expressed in *Nicotiana benthamiana*, revealing that their presence was beneficial to infection. Co-expression of PiRXLRs that target and suppress different immune pathways resulted in an additive enhancement of colonization, indicating the potential to study effector combinations using transient expression assays. We provide a broad platform of high confidence *P. infestans* effector candidates from which to investigate the mechanisms, singly and in combination, by which this pathogen causes disease.

## Teaser

Fifty-two infection-induced *Phytophthora infestans* RXLR effectors have diverse subcellular localizations, and the majority significantly enhance infection when expressed in leaves. Combining effectors targeting different defence pathways additively enhances colonization.

## Introduction

Oomycetes are amongst the most successful groups of plant pathogens. They cause destructive diseases of economically important crops (Kamoun *et al.*, 2015). For example, *Phytophthora sojae* causes severe damage to soybean, *P. ramorum* is responsible for Sudden Oak Death, *P. palmivora* infects a wide range of tropical plants, and *Peronosclerospora sorghi* causes disease on sorghum ( Rizzo *et al.*, 2005; Tyler, 2007; Perumal *et al.*, 2008; Torres *et al.*, 2016). *Phytophthora infestans*, the first characterized pathogenic oomycete species, causes destructive yield losses worldwide in potato and tomato (Fry *et al.*, 2015). Research on these phytopathogens over the last decade has been focused on understanding their molecular mechanisms of pathogenesis (Anderson *et al.*, 2015; Whisson *et al.*, 2016).

Many filamentous pathogens exert their virulence through effector proteins which are thought to promote pathogen colonization by modulating plant innate immunity (e.g. Whisson *et al.*, 2016; Lanver *et al.*, 2017). *Phytophthora infestans* produces both cytoplasmic and apoplastic effectors. Apoplastic effectors act outside the plant cell and include inhibitors of host hydrolases and proteases (Tian *et al.*, 2007; Damasceno *et al.*, 2008), whereas cytoplasmic effectors function within host cells and have a diverse range of targets and activities (Whisson *et al.*, 2016).

The study of oomycete cytoplasmic effectors was launched by the discovery of the conserved RXLR motif (Rehmany *et al.*, 2005). This is a highly conserved Arg–any amino acid–Leu–Arg (RXLR) peptide motif that is required for these effectors to be translocated from the pathogen into plant cells (Whisson *et al.*, 2007; Dou *et al.*, 2008), and has been found in effectors from a range of oomycete species (McGowan and Fitzpatrick, 2017). Numerous candidate RXLR effector genes from *Phytophthora* species have been predicted using bioinformatic screens: >500 in *P. infestans*; ~374 from *P. ramorum*; and 396 from *P. sojae* (Jiang *et al.*, 2008; Haas *et al.*, 2009). Transcripts from >245 RXLR genes were detected during early stages of infection by *P. infestans* (Yin *et al.*, 2017), indicating that many may act in concert to suppress host immunity. Indeed, several RXLR effectors have been shown to suppress distinct plant immune pathways (e.g. Anderson *et al.*, 2015; Whisson *et al.*, 2016).

Amongst the best-studied oomycete RXLR effectors is AVR3a, from *P. infestans*, which is recognized by the potato resistance protein R3a (Armstrong *et al.*, 2005). AVR3a can suppress the cell death induced by the elicitin INF1 (Bos *et al.*, 2006), a pathogen-associated molecular pattern (PAMP). AVRblb2 significantly enhances susceptibility of host plants to *P. infestans* by preventing secretion of the host papain-like cysteine protease C14 at the haustorial interface (Bozkurt *et al.*, 2011). Avr3b from *P. sojae* may act as a Nudix hydrolase in plant cells to impair host immunity (Dong *et al.*, 2011). ATR1 and ATR13 identified in *Hyaloperanospora arabidopsidis* confer enhanced virulence when expressed inside host cells (Sohn *et al.*, 2007). For the majority of RXLR effectors, their biological activities and molecular mechanisms are unknown.

The localization of plant pathogen effectors is crucial for their virulence function (Schornack *et al.*, 2010; Hicks and Galán, 2013; McLellan *et al.*, 2013). Bacterial effectors have been localized to a variety of eukaryotic plant cell compartments; mainly the cytoplasm, nucleus, and plasma membrane (PM), with very few associated with the cytoskeleton, mitochondria, and chloroplasts (e.g. Choi *et al.*, 2017). This defined spatial and temporal context is often required for biochemical activities and virulence (Hicks and Galán, 2013).

Direct observation of effector translocation from oomycete pathogens to plant cells is challenging. One reason is that the transformation efficiency of most oomycete species is very low and recovery of transformants with a high level of expression of transgenic candidate effectors is rare. The amount of effector translocated also appears to be low and for *P. infestans* AVR3a, the translocated fluorescent signal was diluted in the plant cell cytoplasm (Whisson *et al.*, 2007; Boevink *et al.*, 2011). Therefore, *Agrobacterium tumefaciens*-mediated transient expression of fluorescent-tagged candidate pathogen effectors has been employed to determine their subcellular localization (Bos *et al.*, 2010; Schornack *et al.*, 2010; Boevink *et al.*, 2011; McLellan *et al.*, 2013; Whisson *et al.*, 2016). This method was used for subcellular localization of RXLR effectors from *H. arabidopsidis*, which revealed that the host nucleus is a commonly targeted compartment. In addition, the membrane network and cytoplasm are important effector locations. For example, a tonoplast-localized *H. arabidopsidis* effector was shown to enhance plant susceptibility (Caillaud *et al.*, 2012). A few well-studied RXLR effectors from *P. infestans* (PiRXLRs) have been shown to localize to a variety of plant cell compartments; for example, AVR3a localizes to the cytoplasm and nucleoplasm (Bos *et al.*, 2010); Pi04314 localizes to the nucleus and nucleolus, with cytoplasmic background (Boevink *et al.*, 2016*a*); Pi03192 associates with the endoplasmic reticulum (ER) (McLellan *et al.*, 2013); and PexRD54 has been observed to associate with autophagosomes (Dagdas *et al.*, 2016). To date, the subcellular locations of 19 PiRXLRs have been determined (Supplementary Table S1 at *JXB* online). The majority of the RXLR effector repertoire of *P. infestans* is uncharacterized.

Previous studies identified many *P. infestans* RXLR effector candidates by identifying the pathogen transcripts that were highly expressed during infection (Whisson *et al.*, 2007; Haas *et al.*, 2009; Oh *et al.*, 2009; Cooke *et al.*, 2012; Yin *et al.*, 2017). Here, we prioritized 52 PiRXLR effector candidates (PiRXLRs) detectably expressed during infection. Green fluorescent protein (GFP)-tagged effectors were transiently expressed in the model plant *N. benthamiana* for localization or in combination with *P. infestans* to determine their impact on the rate of infection. Most effectors localized to the cytoplasm, nucleus, or PM; effector subcellular localization provides critical clues for understanding virulence functions in plant cells. Using the *N. benthamiana–P.infestans* pathosystem, which has been widely used for host–*P. infestans* interaction studies (Whisson *et al.*, 2016), we found that transient expression of the majority of PiRXLR effector candidates promoted *P. infestans* colonization. Several *P. infestans* effectors have been shown specifically to inhibit defence signalling pathways (e.g. Bos *et al.*, 2010; King *et al.*, 2014; Zheng *et al.*, 2014; Boevink *et al.*, 2016*a*; Yang *et al.*, 2016; Turnbull *et al.*, 2017; Murphy *et al.*, 2018). Effectors that inhibit either the same or different pathways were tested in combination, and the latter provided an additive enhancement to *P. infestans* infection.

## Materials and methods

### Vector construction and *Agrobacterium tumefaciens* transient assays (ATTAs)

Candidate *P. infestans* RXLR effectors were cloned or synthesized without their predicted signal peptides (SPs). For cloning, sequences were amplified from genomic DNA of isolate 88069, using PCR with gene-specific primers including flanking Gateway^®^ recombination sites at both ends (primer sequences are provided in Supplementary Table S2). To generate entry clones, PCR products were purified, then recombined using the Gateway^®^ system into pDONR201 (Invitrogen). Twenty-two effector sequences listed in Supplementary Table S3 were synthesized (Genscript) and provided in the vector pUC57 (Rietman *et al.*, 2012). Effector entry clones were recombined with destination vectors pB7WGF2, pB7WGR2 (Karimi *et al.*, 2005), or a modified pMDC43, with a SP and monomeric red fluorescent protein (mRFP) replacing the GFP sequence, using the LR recombination reaction. Completed clones were electroporated into *A. tumefaciens* strains AGL1 or GV3101 for transient expression of fusion proteins *in planta*. Transformed *A. tumefaciens* strains were confirmed using colony PCR, and were cultured overnight with shaking in yeast-extract and beef (YEB) medium at 28 °C with selective antibiotics. Bacteria from aliquots of the cultures were pelleted and resuspended in infiltration buffer (10 mM MES, 10 mM MgCl_2_, and 200 mM acetosyringone, pH 7.5) to a suitable final concentration as measured by the absorbance at 600 nm (OD_600_). The OD_600_ values used were: 0.1 for effector constructs for individual effector virulence testing, 0.5 for western blotting and hypersensitive response (HR) analysis, 0.01–0.1 for confocal imaging, 0.05 for each effector construct for effector combinations, and 0.05 for the comparative individual effector virulence test with the free GFP control, empty pB7WGF2, used in place of a second effector. Suspensions were incubated at room temperature in the dark for at least 2 h prior to infiltration into leaves.

ATTAs were conducted essentially as described by Kunjeti *et al.* (2016). Briefly, three middle leaves were used from 4-week-old *N. benthamiana* plants, which were grown in a controlled environment in a glasshouse at 22 °C with a 16 h photoperiod and 55% humidity. *Agrobacterium tumefaciens* carrying plasmids to express mRFP or GFP (empty pB7WGF2, or pB7WGR2 respectively) were infiltrated into the air spaces of one half of the mid-vein of each leaf as a control, and bacteria containing the same vector carrying an effector into the other half. On the second day, zoospores from *P. infestans* wild-type strain 88069, cultured as described in Grenville-Briggs *et al.* (2005), were collected for plant infection. On each infiltrated site, 10 µl of zoospores (50000 zoospores ml^–1^) were applied. Lesion diameter was measured at 7 days post-infiltation (dpi) (McLellan *et al.*, 2013). Each leaf typically had four inoculation sites, and 18 leaves were used for each of three replicates (*n*=108 per construct). Boxplots present lesion diameter relative to that for the control. A one-way ANOVA test was performed to identify statistically significant differences.

Cell death assays were performed by infiltrating agrobacteria containing an INF1 construct or co-infiltrating agrobacteria containing Cf4 and Avr4 constructs at an OD_600_=0.5 and were recorded between 4 and 8 dpi as previously described (Gilroy *et al.*, 2011), using one-way ANOVA to assess statistical significance.

### Immunoprecipitation and western analysis

Three-week-old *N. benthamiana* middle leaves were infiltrated with *A. tumefasciens*-containing constructs to express GFP- or RFP-tagged RXLR effector fusion proteins. Leaf discs of 1 cm in diameter were collected after 2 d, and were ground in liquid nitrogen (LN_2_) and resuspended in 100 µl of GTEN buffer, described in Boevink *et al.* (2016*b*). Samples were centrifuged at 17000 *g* for 10 min at 4 °C. For immunoprecipitation of Pi04314–mRFP, mRFP–Trap _M beads (Chromotek) were used to capture fusion proteins according to the manufacturer’s instructions. For western immunoblot analysis, 100 µl of supernatant was mixed with the same volume of 2× SDS–PAGE sample loading buffer [100 mM Tris–HCl (pH 6.8), 200 mM DTT, 4% (w/v) SDS, 0.2% (w/v) bromophenol blue, 20% (v/v) glycerol]. Samples (10 µl) were loaded onto a 10% Bis-Tris SDS–PAGE gel. The gel was run with 1× Tris running buffer [10× stock: 250 mM Tris base, 1.92 M glycine, 1% (w/v) SDS] for 30 min at 80 V, then at 110 V for another 2 h. Gels were electroblotted onto a nitrocellulose membrane for 1.5 h at 30 V, and Ponceau staining, membrane blocking, and washing steps were carried out as described by McLellan *et al.* (2013). The αmRFP and αGFP primary antibodies (Chromotek) were used at 1:4000 and 1:1000 dilutions, respectively. Secondary antibodies anti-rat IgG–horseradish peroxidase (HRP; Chromotek) or anti-mouse IgG–HRP (Sigma-Aldrich) were used at 1:5000 dilutions. Protein bands on immunoblots were labelled with ECL substrate (Thermo Scientific Pierce) using the manufacturer’s protocol and imaged with Amersham Hyperfilm™ ECL, developed with a Xograph imaging system, compact X4 developer.

### Confocal imaging

Leaf cells transiently expressing effector fusions were imaged 24–72 h post-infiltration on Leica SP2, Zeiss 710, or Nikon A1R confocal microscopes using water dipping objectives. GFP was excited with 488 nm light and the emissions were detected between 500 nm and 530 nm. mRFP, mCherry, and tdTomato fluorescent proteins were excited with 561 nm light and their emissions detected between 600 nm and 630 nm for the first two fluorophores and 590–620 nm for tdTomato (on the Leica SP2 and Zeiss 710; on the Nikon A1R the filter for all red fluorophores provided a window of 570–620 nm). Cyan fluorescent protein (CFP) was excited with 405 nm light and emissions were detected between 455 nm and 480 on the Zeiss 710. Yellow fluorescent protein (YFP) was excited with 514 nm light and emissions were detected between 520 nm and 550 nm. The pinhole was set at 1 Airy unit for the longest wavelength fluorophore of any combination. Effector fusions were examined on multiple occasions on independently infiltrated plants. Cells distributed around infiltration areas displaying a range of protein expression levels were examined at several different magnifications for each effector fusion construct. Generally cells displaying a low level of fluorescence were imaged to minimize overexpression artefacts. For co-expression analyses, the *A. tumefaciens* suspensions were pre-mixed before infiltration. Co-expression experiments were performed on independent occasions, and cells distributed around infiltration areas displaying a range of protein expression levels were examined at different magnifications. For co-expression with infection by the *P. infestans* tdTomato transformant, the transformant was inoculated onto leaves and the infection was allowed to develop for 2–3 d before agroinfiltration was applied. The frequency of cells that were both transiently expressing the agrobacteria-derived fusion proteins to a suitable level for imaging and infected with the transformant at an early stage so as not to show cellular disruption was low.

The commonly used organellar marker tags ST–mRFP, mRFP–SRL, mRFP–ATG8, Ara6–mRFP, and mRFP–Ara7 are described in Engelhardt *et al.* (2012). Nucleoplasmic and nucleolar markers were mRFP-tagged *N. benthamiana* histone 2B (mRFP–H2B) and mRFP-tagged *A. thaliana* Fibrillarin1 (mRFP–Fib1), respectively (Goodin *et al.*, 2007). mCherry–TUA5 was used to label the microtubules (Gutierrez *et al.*, 2009; Eisinger *et al.*, 2012). GFP fused to LTi6b was used to label the PM (Kurup *et al.*, 2005). The potato orthologue of the exocyst component Sec5, StSec5, was amplified using primers with attB recombination sites (Supplementary Table S2) and recombined into pDONR201 before recombination into the destination vector pB7WGY2 to create a YFP-tagged form (Karimi *et al.*, 2005). MitoTracker red dye (Thermo-Fisher Scientific) was used to label mitochondria. Following infiltration into leaves at a concentration of 0.1 μM, the leaves were washed with water and incubated for a minimum of 1 h as it was found that a longer incubation time resulted in more specific labelling.

In leaves co-expressing effector fusions and organellar markers or infected with tdTomato-expressing *P. infestans*, the fluorophores were imaged sequentially to minimize cross-talk. Images were processed with propriety confocal software or ImageJ as required. Figures were constructed with Adobe Photoshop and Adobe Illustrator.

## Results

### GFP–RXLR effector fusions display a range of subcellular locations

**Fig. 1.**
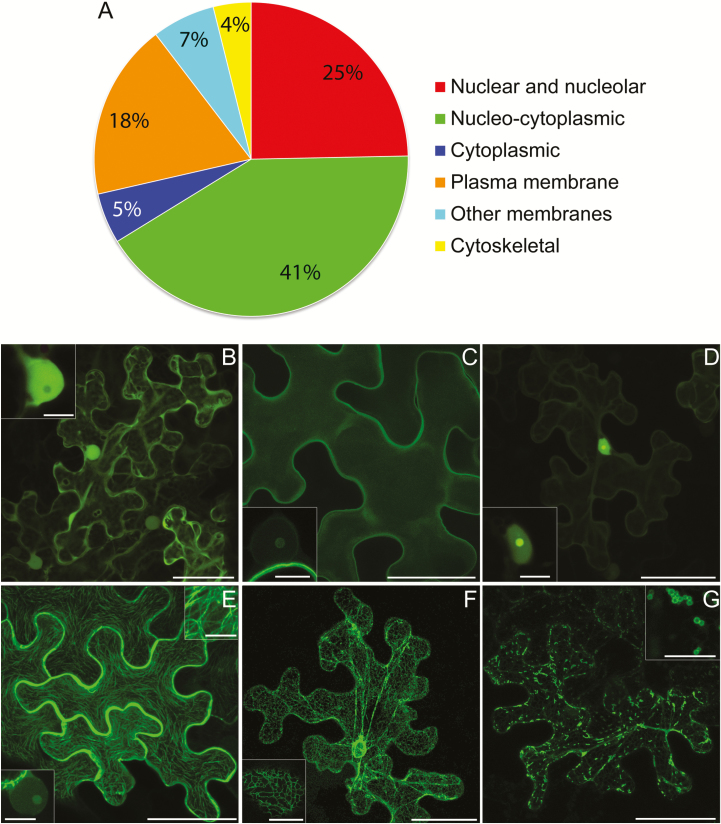
Summary and examples of localization patterns for transiently expressed effector fusions. A pie chart representation of the main localization patterns of GFP–effector fusions examined in this work combined with those previously published (A). The distinction between nucleo-cytoplasmic and cytoplasmic is that in the latter there was little or no fluorescence in the nucleus. The confocal projection images (B–D) show the most abundant patterns: nucleo-cytoplasmic (B), plasma membrane (C), and association with the nucleus (D). The insets show single optical sections through nuclei. Several of the plasma membrane-localized fusions had detectable fluorescence in the nucleolus (inset in C). All of the nuclear-associated fusions displayed some level of nucleolar labelling (inset in D). The images (E–G) represent some other patterns of localization observed for RXLR effector fusions. These were association with the microtubule cytoskeleton (E), two of which also labelled the nucleolus (lower inset in E), endoplasmic reticulum (F), and mitochondria (G; insets show higher magnification images). Scale bars are 50 μm for the main images and 10 μm for the insets.

Fifty-two PiRXLR effector candidates were selected based on those reported to be induced during infection (Supplementary Table S1; Whisson *et al.*, 2007; Haas *et al.*, 2009; Cooke *et al.*, 2012; Ah-Fong *et al.*, 2017; Yin *et al.*, 2017). Previous studies have suggested that the C-termini of RXLR proteins are important for their effector functions (e.g. Bos *et al.*, 2006). Therefore, the effector candidates were tagged with GFP at their N-termini, following removal of the SP, to avoid interfering with the C-terminal effector domain (ED), and transiently expressed in *N. benthamiana* for assessment of their subcellular locations. The stability of each construct was confirmed by immunoblotting (Supplementary Fig. S1). The effector fusion localizations were categorized based on the most commonly observed or dominant pattern of fluorescence compared with or expressed with standard cellular markers. The localizations observed are summarized in Fig. 1A and example images of the most common patterns, nucleo-cytoplasmic, PM, and nuclear, are shown in Fig. 1B–D. Example images representing additional localization patterns are shown in Fig. 1E–G. Several effector fusions showed variation in their subcellular or subnuclear fluorescence patterns as noted in Supplementary Table S1 and the legends for Supplementary Figs S2–4. Pi18215 was too variable to place it in a particular category.

Combining the 52 effector fusions examined here and the 19 previously published effector localizations in *N. benthamiana*, in all 32 were either predominantly cytoplasmic or nucleo-cytoplasmic (Fig. 1B; Supplementary Table S1). Supplementary Fig. S2 shows typical images for the effector fusions characterized here that showed cytoplasmic and nucleo-cytoplasmic patterns of fluorescence. Images of untagged, nucleo-cytoplasmic GFP and the PM-localized mOrange–LTi6b fusion are included for comparison. In total, 15 effector fusions appeared to be associated with the PM, 12 of which were characterized here (Supplementary Fig. S3), and nine of these were also detectable in the host nucleus, in all cases showing a degree of nucleolar labelling (Fig. 1C; Supplementary Table S1). Nineteen effector fusions demonstrated strong nuclear association, 14 of which were characterized here and are shown in Supplementary Fig. S4A. All of them were co-expressed with nuclear markers, and two examples of co-expression are shown in Supplementary Fig. S4B. All showed some nucleolar fluorescence (Fig. 1D; Supplementary Table S1). Two effectors, Pi07550 and Pi09732, that initially appeared to be nucelo-cytoplasmic were found by western blotting to be unstable as GFP fusions (results not shown), and thus the apparent localizations represented only the unfused GFP. These effectors were re-cloned as just the ED (from after the RXLR-EER domain to the C-terminus) and fused to GFP. In this form they were stable (Supplementary Fig. S1), and fluorescence was predominantly located in the nucleus for GFP–Pi07550 ED and at the PM for GFP–Pi09732 ED (Supplementary Table S1; Supplementary Figs S3, S4).

Three effector fusions, GFP–Pi07387, GFP–Pi14788, and GFP–Pi15110, associated with the microtubule cytoskeleton (Fig. 1E; Supplementary Table S1). This was confirmed by co-expression with mCherry-tagged tubulin 5 (TUA5) (Supplementary Fig. S5). Faint nucleolar fluorescence was also observed for GFP–Pi14788, while GFP–Pi07387 (Avr4) was strongly associated with the nucleus (Supplementary Fig. S4) and was not associated with microtubules in every cell. GFP–Pi09218 associated with mitochondria, and faint fluorescence was also observed at the ER (Fig. 1G; Supplementary Table S1; Supplementary Fig. S5). Mitochondrial labelling was confirmed by co-labelling with MitoTracker Red (Thermo Fisher Scientific). GFP–Pi04049 showed nucleo-cytoplasmic fluorescence combined with small mobile bodies. To attempt to identify these bodies, the effector fusion was co-expressed with a variety of markers, but none showed co-localization (Supplementary Fig. S6).

**Fig. 2.**
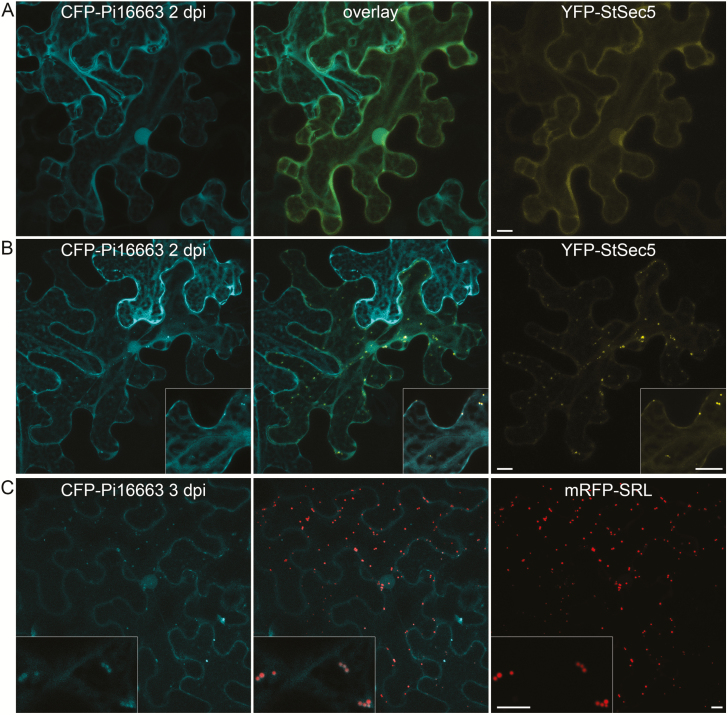
Effector Pi16663 co-localizes with YFP–StSec5 and peroxisomes. Transiently co-expressed CFP–Pi16663 and YFP–StSec5 are generally nucleo-cytoplasmic in some cells (A) and, in cells displaying mobile bodies labelled by YFP–StSec5, the CFP–Pi16663 is also associated with them (B). At 3 dpi, CFP–Pi16663 localizes to small mobile bodies that co-label with the peroxisome marker mRFP–SRL (C). Inset images are at higher magnification. Scale bars represent 10 μm.

Fluorescent protein-tagged Pi16663 (Avr1) was nucleo-cytoplasmically localized at 2 dpi (Fig. 2A; Supplementary Figs S2, S7A). This effector has been shown to interact with the host protein Sec5 (Du *et al.*, 2015*a*). Transiently expressed YFP–StSec5 from potato is nucleo-cytoplasmic and, in some cells, also locates to small mobile bodies. Upon co-expression with YFP–StSec5 at 2 dpi, CFP–Pi16663 co-localized in the cytoplasm and at mobile bodies, when they were present, which is consistent with an interaction between these proteins (Fig. 2A, B). Curiously, at 3 dpi, CFP–Pi16663 expressed alone labels peroxisomes (Fig. 2C). At 2 dpi, CFP–Pi16663 is not associated with peroxisomes (Supplementary Fig. S7A). Moreover, YFP–StSec5-labelled bodies do not co-localize with the peroxisome marker (Supplementary Fig. S7B). Co-expression of the YFP–StSec5 at 3 dpi appeared to reduce the association of CFP–Pi16663 with peroxisomes (Supplementary Fig. S7C).

### Few effector fusion proteins accumulate around haustoria

**Fig. 3.**
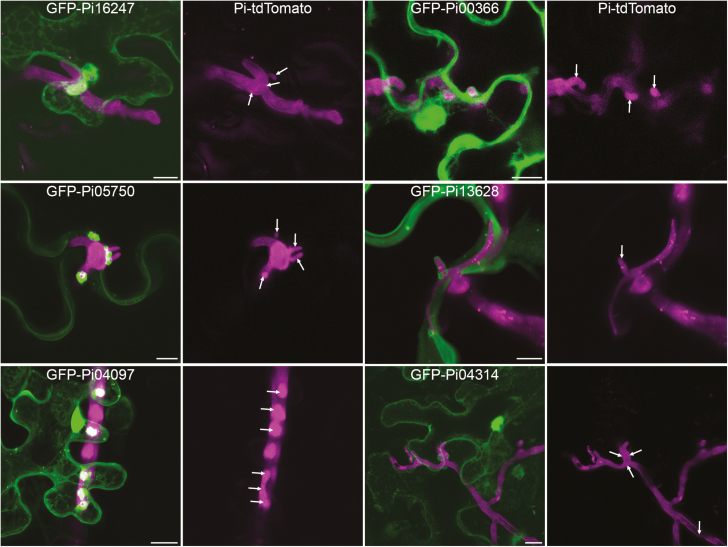
Some effector fusions accumulate around haustoria. Leaves infected with tdTomato-expressing *P. infestans* were infiltrated with agrobacteria containing plasmid constructs to express effector fusions transiently. Confocal projection images show cells that are penetrated by haustoria and also expressing effector fusions. Only cells that showed normal subcellular organization were imaged. The left panel shows examples of effector fusions whose main localization was nucleo-cytoplasmic, plasma membrane, and nuclear (from top to bottom) that did accumulate around haustoria. For comparison, the right panel shows effector fusions with the same localizations that did not accumulate around haustoria. The magenta-only panels are included to show the hyphae and haustoria, though many haustoria were either facing the lens or very small and thus cannot be distinguished from the hyphal fluorescence. Haustoria are indicated with arrows. Scale bars represent 10 μm.

The behaviour of *in planta* expressed effector fusions in cells infected by *P. infestans* was examined, to determine if any of them particularly accumulated around haustorial penetration sites as described for Avrblb2 (Bozkurt *et al.*, 2011) and Avr2 (Saunders *et al.*, 2012). The results are summarized in Supplementary Table S1. Of the effectors examined here, suitable cells were found for expression of 25 effectors with *P. infestans* infection combinations. Of these, seven appeared to accumulate strongly around haustoria compared with others showing the same localization patterns. In total 18 did not appear to accumulate particularly around haustoria. Figure 3 shows example images of nucleo-cytoplasmic, PM, and nuclear GFP–effector fusions that either do or do not accumulate at haustoria. Given that accumulation at haustoria does not appear to be a general property of RXLR effectors, it is likely that any observed accumulation is due to the behaviour of the effector targets. Pi16663 (Avr1), for example, associates with both peroxisomes and Sec5-associated subcellular bodies. Both of these types of subcellular bodies can be observed clustering around haustoria (Supplementary Fig. S8), probably explaining why GFP–Pi16663 (Avr1) accumulates at haustoria.

The AvrBlb2 family member Pi04097 (SFI1) localizes strongly in the nucleus and accumulates around haustoria (Supplementary Table S1; Fig. 3). Previously, we have shown that if its nuclear localization is attenuated by addition of a myristoylation signal to the N-terminus (myrGFP–Pi04097), it fails to suppress flg22-triggered immunity or enhance *P. infestans* colonization (Zheng *et al.*, 2014). However, we show here that expression of myrGFP–Pi04097 results in a Blb2-mediated hypersensitive response, indicating that recognition by this resistance protein does not require nuclear localization (Supplementary Fig. S9).

### Two RXLR effectors enhance *P. infestans* colonization only when they are expressed inside plant cells

**Fig. 4.**
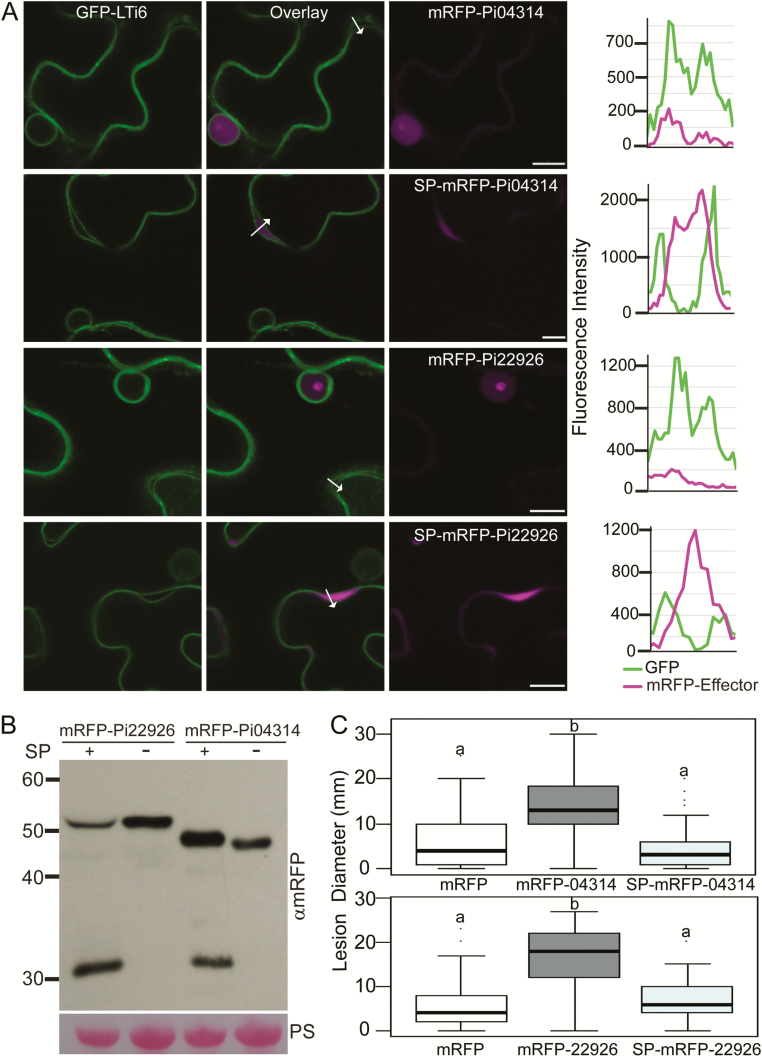
RXLR effectors function inside plant cells. Single optical section confocal images of N-terminally tagged effector fusion proteins expressed in transgenic *N. benthamiana* in which the plasma membrane and nuclear membrane were labelled with GFP–LTi6b (A). mRFP–Pi04314 localizes to the nucleus and nucleolus. SP–mRFP–Pi04314 was secreted from the plant cell into the apoplast and was not observed inside the cells (upper two panels). The localization of Pi22926 fusions with and without signal peptide were the same as for Pi04314 (lower two panels). The arrows indicate paths used for the fluorescence intensity profiles of mRFP and GFP across the plasma membranes and apoplast of adjoining cells; the profiles are shown at the right of the image sets. The *x*-axes represent the lengths of the arrows. Regions where the plasma membranes of adjoining cells were slightly parted were chosen for clarity. Scale bars indicate 10 µm. (B) Immunoblots of the constructs (mRFP–Pi04314, SP–mRFP–Pi04314, mRFP–Pi22926, and SP–mRFP–Pi22926) expressed on *N. benthamiana* show the stability of the fusion proteins using an mRFP antibody. Free mRFP was also observed when effector fusions were secreted. The size marker is indicated in kDa, and protein loading is indicated by Ponceau stain (PS). (C) *P. infestans* colonization of *N. benthamiana* increased significantly following *Agrobacterium*-mediated expression of mRFP–Pi04314 and mRFP–Pi22926 compared with free mRFP, but not following expression of SP–mRFP–Pi04314 or SP–mRFP–Pi22926. Boxplots represent the combined data from three biological replicates (*n*=108 per construct). Letters on the boxplots denote statistically significant differences (ANOVA, *P*<0.001).

To confirm whether the presence of RXLR effectors inside plant cells is essential for them to benefit infection, two RXLR effectors, Pi04314 and Pi22926, were selected for expression with and without native SPs *in planta*. mRFP was fused to the N-termini of Pi04314 and Pi22926. Both mRFP–Pi04314 and mRFP–Pi22926 accumulated in the nucleus and nucleolus when transiently expressed without a SP in *N. benthamiana* (Fig. 4A, B) same as previous observations with their C terminal tag (Wang et al., 2018,Boevink et al., 2016a), and both enhanced *P. infestans* colonization significantly (Fig. 4C), as shown previously for Pi04314 (Boevink *et al.*, 2016*a*; S. Wang *et al.*, 2017). Moreover, mRFP–Pi04314 is able to co-immunoprecipitate its published target StPP1c (Boevink *et al.*, 2016*a*) (Supplementary Fig. S10A). In contrast, the same effectors expressed with an SP were observed to be secreted into the plant apoplastic space (Fig. 4A, B) in the same manner as SP–mRFP (Supplementary Fig. S10B), and failed to enhance *P. infestans* colonization (Fig. 4C), confirming earlier findings that RXLR effectors are active only when expressed inside the plant cell.

### Most RXLR effectors enhance *P. infestans* colonization

**Fig. 5.**
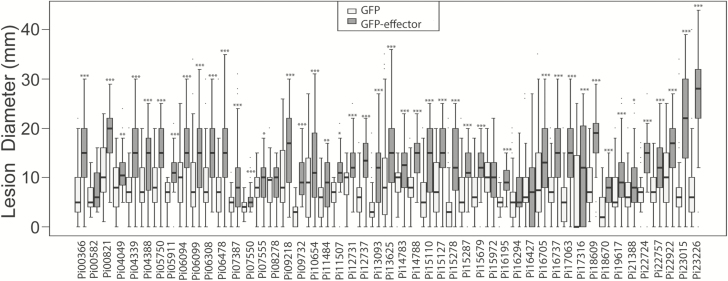
Virulence test of *P. infestans* RXLR candidate effectors. *Agrobacterium tumefaciens* transient assays (ATTAs) were performed to determine the impact of the expression of N-terminally GFP-tagged RXLR effectors on colonization of *P. infestans.* The effector fusions were compared with free GFP expressed from the same vector. Of the 51 candidate RXLR effectors tested in this experiment, 44 boosted the growth of *P. infestans* significantly (as indicated with asterisks) while five effectors did not. Two effectors are not shown as they caused cell death. *P*-values were from a one-way Student’s *t*-test (*P<0.05, **P<0.01,***P≤0.001). Each effector is represented by a minimum of 72 replicates.

The effector genes, minus SP sequences, were expressed transiently and individually in *N. benthamiana* to test whether they are able to enhance *P. infestans* infection. Using *A. tumefaciens,* the GFP-tagged effectors were expressed in one half of *N. benthamiana* leaves, with free GFP expressed in the other half as a control. The infiltrated areas were subsequently infected with *P. infestans* and the lesion sizes were measured at 7 dpi. Statistical analysis of the *P. infestans* lesion diameter data in the ATTA indicated that, compared with the expression of free GFP control, 45 of the 52 effectors tested significantly enhanced *P. infestans* colonization (Figs 4, 5; Supplementary Table S1). Five of the RXLR effector candidates produced stable proteins when expressed transiently in *N. benthamiana* but did not produce a statistically significant enhancement of *P. infestans* colonization: Pi16294 (AvrVnt1), Pi15972, Pi16427, Pi08278, and Pi00582 (Fig. 5). In addition, two effectors, Pi08174 and Pi10232, triggered cell death strongly in *N. benthamiana* leaves (Supplementary Fig. S11) and so could not be assessed. The cell death response suggests that either they were recognized, potentially by R proteins, to trigger an immune response in *N. benthamiana*, or their presence in excess was toxic to plant cells.

### Co-expressing effectors can have additive effects

RXLR effectors can suppress plant immunity by manipulating different signalling pathways. Some effectors interfere with the early stages of flg22-triggered transcript accumulation, such as Pi06087 (SFI3/PexRD16) and Pi09585 (SFI4) (Zheng *et al.*, 2014). Both of these effectors enhance colonization by *P. infestans* when transiently expressed in *N. benthamiana* (Zheng *et al.*, 2014), and have been shown to be highly up-regulated at the early stages of both leaf (Haas *et al.*, 2009) and tuber (Ah-Fong *et al.*, 2017) infection and in diverse pathogen genotypes (Yin *et al.*, 2017). Other effectors, such as Pi02860 and Pi11383 (PexRD2), inhibit programmed cell death triggered by distinct elicitors. Pi02860 suppresses INF1-triggered cell death, but not cell death triggered by co-expression of *Cladosporium fulvum* Avr4 with the tomato Cf4 resistance protein (Yang *et al.*, 2016). In contrast, Pi11383 (PexRD2) suppresses Avr4/Cf4-mediated cell death but not INF1-triggered cell death (King *et al.*, 2014). Whilst *P. infestans* lacks the flg22 MAMP and does not produce the *C. fulvum* Avr4 protein, the pathways activated by perception of these proteins are generic and are probably activated by detection of unknown *P. infestans* molecules by uncharacterized receptors. Indeed, the recently characterized receptor StLRPK1, which provides resistance to *P. infestans*, activates the same signalling pathway as Cf4 (H. Wang et al., 2018).

**Fig. 6.**
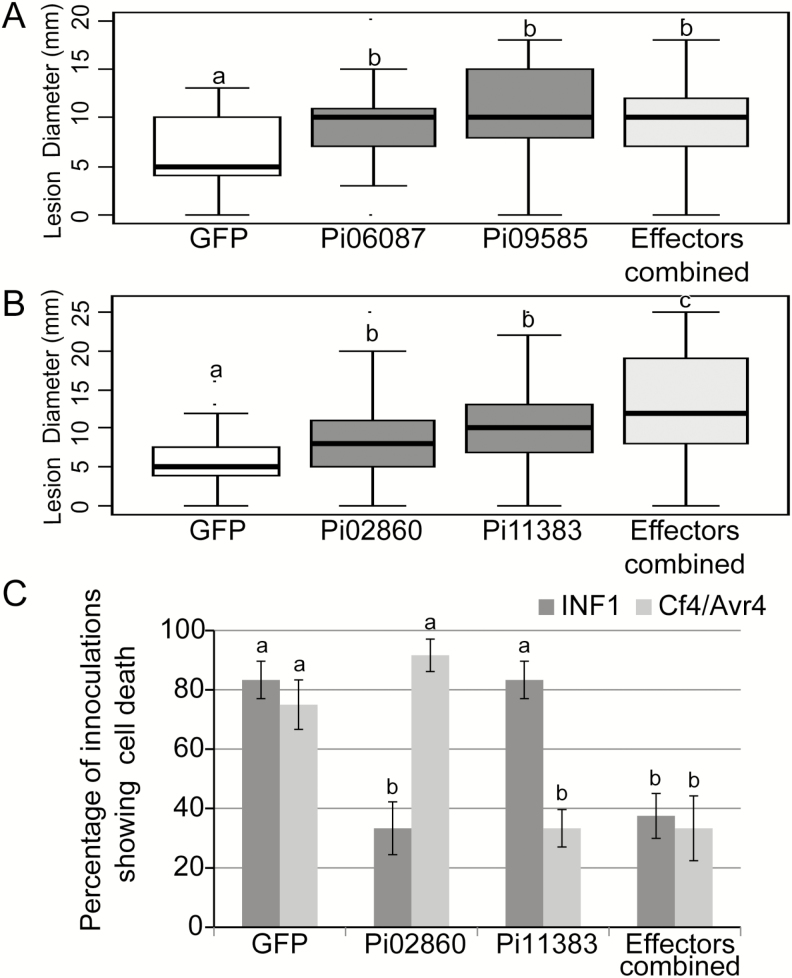
Virulence tests with combinations with *P. infestans* RXLR effectors. Transient co-expression of RXLR effectors Pi06087, Pi09585, Pi02860, and Pi11383 with N-terminal GFP tags significantly enhanced *P. infestans* colonization individually compared with the GFP vector control (A, B). Co-expression of Pi06087 and Pi09585 did not provide an increased effect on *P. infestans* colonization compared with the effectors alone (A). The co-expression of Pi02860 and Pi11383, however, did provide an increased effect on *P. infestans* colonization (B). Transient expression of Pi02860 and Pi11383 could suppress cell death triggered by transient co-expression of INF1 or Cf4 and Avr4, respectively (C). Co-expression of the two effectors suppressed cell death triggered by both INF1 and Cf4/Avr4 (C). Boxplots represent the combined data from three biological replicates (*n*=79 per construct). Letters a, b, and c on the boxplots and graph denote statistically significant differences (ANOVA, *P*<0.001).

Pairs of effectors that inhibit either the same or different signalling pathways were selected to assess whether they would have an additive effect in terms of enhancing *P. infestans* colonization. The co-expression of Pi06087 (SFI3/PexRD16) and Pi09585 (SFI4), which both inhibit the flg22-triggered signalling pathway, did not show an additive effect on infection compared with the enhanced colonization observed following expression of each of these effectors individually (Fig. 6A). In contrast, co-expression of Pi02860 and Pi11383 (PexRD2), which suppress different defence signalling pathways, showed an enhancement of infection greater than that of either effector expressed alone (Fig. 6B). Critically, the combined expression of these effectors suppressed both INF1- and CF4-triggered cell death pathways, whereas each individual effector alone could only suppress one immune pathway (Fig. 6C).

To extend our analyses, we selected for co-expression additional effector combinations that suppress distinct immune pathways. Whereas Pi13628 (SFI5) specifically suppresses flg22-triggered signalling (Zheng *et al.*, 2014), Pi11383 (RD2) suppresses only Cf4-triggered cell death (King *et al.*, 2014), and Pi17316 specifically suppresses INF1-triggered cell death (Murphy *et al.*, 2018). Combinations of pairs of these effectors in all cases provided an additive enhancement of colonization compared with each effector alone (Supplementary Fig. S12A). In contrast, any pairwise combinations of Pi02860, Pi17316, or PiAvr2, all of which suppress INF1-triggered cell death (Yang *et al.*, 2016; Turnbull *et al.*, 2017; Murphy *et al.*, 2018), provided no additive enhancement of colonization beyond each effector alone (Supplementary Fig. S12B).

## Discussion

Cytoplasmic effectors are defined as those that function specifically within host cells, and the past decade of genome sequencing has seen the identification of hundreds of candidate cytoplasmic effectors in plant pathogen genomes. Information on where those effectors localize within plant cells and whether their presence there benefits pathogen infection are critical next steps in prioritizing them for a more detailed functional study. The genome sequence of *P. infestans* reveals >550 candidate RXLR (PiRXLR) effector genes that are predicted to be delivered into plant cells (Haas *et al.*, 2009). Studies using RT-PCR (e.g. Whisson *et al.*, 2007; Oh *et al.*, 2009; Zheng *et al.*, 2014), microarrays (Haas *et al.*, 2009; Cooke *et al.*, 2012), and RNA sequencing (e.g. Ah-Fong *et al.*, 2017; Yin *et al.*, 2017) provide evidence that fewer than half of the PiRXLR genes show detectable characteristic transcript accumulation in the first 2–3 d of infection in various *P. infestans* genotypes. To date, only 19 PiRXLR effector candidates have been expressed in plant cells to determine where they localize and whether they enhance infection by the pathogen. We selected a further 52 PiRXLRs to determine their subcellular localization and whether, following transient expression in the model host *N. benthamiana*, they enhance *P. infestans* leaf colonization (Supplementary Table S1). Our observations are discussed below.

### RXLR effectors show a range of localizations within plant cells

In accordance with the wide variety of known effector activities (reviewed in Whisson *et al.*, 2016), fluorescent protein-tagged effectors display a range of subcellular locations. The three most common locations were the (nucleo-)cytoplasm, nucleus, and PM. Caillaud *et al.* (2012) reported that RXLR effectors from *H. arabidopsidis* were also commonly in the (nucleo-)cytoplasm and nucleus but that membrane-associated effectors were more often associated with the ER than the PM. All other subcellular localization patterns we observed were rare, and it is interesting that while we found three effector fusions that labelled the microtubule cytoskeleton, no effector fusions labelled the actin cytoskeleton. This is perhaps surprising given that the host actin cytoskeleton is known to re-orient towards *Phytophthora* penetration points (Hardham and Blackman, 2010) and is essential to the internalization and signalling of immune receptors (Beck *et al.*, 2012). Similarly, although one effector, Pi09218, associated with mitochondria, none was found to locate to chloroplasts despite the known roles of this organelle in plant defence (Serrano *et al.*, 2016). However, it should be noted that the 71 PiRXLRs (52 studied here and 19 described previously; Supplementary Table S1) for which subcellular localization data exist represent only 43 distinct families out of >150, as determined by TribeMCL Markov clustering (Haas *et al.*, 2009). More extensive analyses of expressed PiRXLR effectors may reveal some that localize to additional sites, such as the actin cytoskeleton or chloroplasts.

The addition of any tag to a protein of interest carries a risk of disrupting signals or interactions; thus some effector localizations may not be correctly displayed. Many effector fusions appear to show degradation products on western blots. If these are not simply non-specific bands or a result of the preparation of material for westerns and do exist within the cells, then some patterns of fluorescence may be due to these. For several published effectors, however, the observed localization is appropriate for their function. For example, Pi03192 locates to the ER and has been shown to interact with ER-located transcription factors (McLellan *et al.*, 2013), whereas Pi04314 localizes to the host nucleus and interacts with PP1c isoforms at that location (Boevink *et al.*, 2016*b*), and Pi09316 (PexRD54) localizes to autophagosomes and interacts with ATG8 (Dagdas *et al.*, 2016).

Functional effector localization may be dependent on the location of the host target with which it associates, and this may only be experimentally observable with co-overexpression. Exocyst component Sec5 is a published interactor of Pi16663 (Avr1; Du *et al.*, 2015*a*). YFP-tagged StSec5 displays nucleo-cytoplasmic fluorescence in some cells and in others it also shows brightly labelled, small, mobile bodies. Fluorescent protein-labelled Pi16663 (Avr1) (FP-Pi16663) was generally nucleo-cytoplasmic at 2 dpi. However, when YFP–StSec5 was co-overexpressed and associated with the small bodies, FP–Pi16663 was also associated with these bodies (Fig. 4). Interestingly, at 3 dpi, the FP–Pi16663 expressed alone associated with peroxisomes (Supplementary Fig. S7). As YFP–StSec5 did not associate with peroxisomes, our observation may indicate that Pi16663 (Avr1) has a second host target. Given that R1 recognition of Pi16663 (Avr1) is dependent on both R1 and the effector being located in the nucleus (Du *et al.*, 2015*b*), a third host target located in the nucleus that is guarded by R1 is a distinct possibility. Similarly PiAvr3a has been shown to interact with a number of host proteins in Y2H, including CMPG1 which is a target for immune suppression (Bos *et al.*, 2010). However, CMPG1 is not a guardee for R3a recognition of PiAvr3a, raising the possibility that one of the other interactors of PiAVR3a may be monitored by R3a.

As indicated above, the 71 PiRXLRs for which subcellular localization has been studied represent 43 families, 14 of which are represented by two or more PiRXLRs (Supplementary Table S1). We can see that members from within a family that are thus related by sequence similarity do not necessarily share the same location. For example, the IPIO4 variant of Pi21388 (IPIO1), which is not present in the sequenced *P. infestans* strain (Champouret *et al.*, 2009), has a strong association with the PM and is greatly reduced in the nucleus compared with the IPIO1 form. Moreover, Pi07387 (Avr4) and Pi22926 are both in RXLRfam52, but only Pi07387 shows association with microtubules. Conversely, the three PiRXLRs that associate with microtubules, Pi07387, Pi14788, and Pi15110, are each members of different families (52, 8, and 1, respectively). It will be interesting to see whether these sequence-unrelated effectors possess similar functions, or are localized to microtubules via association with functionally distinct host proteins.

### Not all RXLR effectors accumulate at haustoria

The site of haustorial development and penetration is likely to be a focus for plant defence activity. It is thus logical that at least some effectors would accumulate around haustoria, perhaps by interacting with components of the plant defence response that are trafficked there. Pi08943 (Avr2) and Pi20300 (AvrBlb2) were previously reported to accumulate at haustoria (Bozkurt *et al.*, 2011; Saunders *et al.*, 2012). It is difficult to obtain useful results from transient co-expression of effector fusions and *P. infestans* infection due to the rarity of locating *P. infestans*-infected cells that also transiently express suitable levels of the effector fusions and the rapid deterioration of cell health upon infection, leading to cellular disruption such as ER and cytoplasmic condensation. Images were thus discarded for effectors where the health of host cells had deteriorated to the point where reliable localization was not possible. Of the 25 effectors for which suitable, healthy cells were found, seven showed noticeably more accumulation around haustoria than other effectors with equivalent subcellular locations (Fig. 3; Supplementary Table S1). The accumulation of effectors at haustoria is likely to be indicative of the accumulation of their host targets at these sites. For example, Pi16663 (Avr1) associated at 2 dpi with Sec5-labelled punctae and later with peroxisomes, and both of these subcellular bodies accumulate around haustoria (Supplementary Fig. S8). Pi04097 (SFI1) is a member of the Avrblb2 family and, as mentioned, Bozkurt *et al.* (2011) reported that Avrblb2 accumulated around haustoria. The Avrblb2 family member described by Bozkurt *et al.* (2011) (Pi20300) was located at the PM, whereas Pi04097 shows an association with the nucleus in both infected and uninfected cells; the accumulation of Pi04097 around haustoria is in addition to its nuclear association in infected cells. The nucleus is an important site for Pi04097 virulence function since its removal from the nucleus resulted in the loss of its ability to enhance *P. infestans* growth (Zheng *et al.*, 2014). This suggests that either Pi04097 has more than one host target, or that a significant proportion of its host target locates at haustoria in addition to the nucleus during infection.

### RXLR effectors are active within cells

Cytoplasmic effectors are thought to function inside plant cells, so studies of effector interactions and activities have been conducted within cells. Here, we provide direct evidence that expression of RXLR effectors within plant cells is essential for them to have an impact on infection. Two effectors were expressed with intact SP: Pi22926 and Pi04314. Neither enhanced *P. infestans* colonization when they were secreted from the plant cells but did when expressed inside the cells without their SP (Fig. 4). Therefore, it is necessary for these effectors to be inside plant cells to function; indeed Pi04314 has been shown to work in the host nucleus (Boevink *et al.*, 2016*b*), and shown previously not to enhance colonization when expressed as an SP–Pi04314–mRFP fusion protein (S. Wang *et al.*, 2017).

The potential virulence function of the 52 selected RXLR effectors in host cells was assessed using ATTAs. Forty-five PiRXLRs enhanced *P. infestans* colonization significantly when expressed inside plant cells (Figs 4, 5). This provides evidence that they are indeed cytoplasmic effectors and that they have important roles in the establishment of infection (Whisson *et al.*, 2016). We interpret the enhancement of infection as likely to be due to having an excess of the effector present inside host cells prior to pathogen perception, leading to the effector being ‘in place’ in sufficient quantity to associate efficiently with it target.

Two of the tested effectors triggered a rapid cell death in this study. Of the 19 previously published RXLR effectors, two triggered cell death (Zheng *et al.*, 2014; H.Y. Wang *et al.*, 2017). How these effectors caused cell death was not characterized. It could be that some are toxic or disruptive to the cells when overexpressed, or they may be recognized by resistance genes in the plant. *Nicotiana benthamiana* is susceptible to *P. infestans*, however, so the possible recognition of these effectors does not lead to resistance. The recognition of these effectors may be suppressed by the action of other effectors. An example of this can be found in the literature. Pi22798-triggered cell death was dependent on SGT1 (suppressor of the G2 allele of *skp1*), suggesting that this is an immune response (H.Y. Wang *et al.*, 2017). This cell death response was suppressed by another *P. infestans* effector, Pi18215 (SFI7, AVR3b) (H.Y. Wang *et al.*, 2017).

Of the other five effectors that failed to promote colonization on *N. benthamiana*, some may only function to mitigate the recognition of other effectors, and potentially are not required to function in *N. benthamiana* due to the absence of that recognition response. It is also possible that the effectors are not fully functional in *N. benthamiana* or as FP fusions. It is interesting that Pi16294 is a known avirulence effector, Avr-vnt1, recognized by an R protein in potato and thus potentially has a function in potato that is important enough to be guarded by an R protein. It may be that some of these effectors are already expressed by the pathogen to a high level during infection and that transient expression does not exceed their natural abundance within the plant cell. Effector Pi14371 (AVR3a) is essential for virulence and is recognized by the potato resistance protein R3a in the cytoplasm (Armstrong *et al.*, 2005; Bos *et al.*, 2010). It interacts with and stabilizes the U-box E3 ligase CMPG1 to block INF1-triggered cell death in *N. benthamiana*, but does not enhance *P. infestans* colonization significantly when transiently expressed (Bos *et al.*, 2010).

For oomycete RXLR effector research on the Arabidopsis pathogen *H. arabidopsidis*, which has a relatively small number of putative RXLR genes that show conservation with those of the *Phytophthora* genus, several high-throughput screens have shown that many affect the plant immune response (Cabral *et al.*, 2011; Anderson *et al.*, 2012; Caillaud *et al.*, 2012; Pel *et al.*, 2014). Few high-throughput screens have been published for *P. infestans* effectors. Zheng *et al.* (2014) previously screened 33 of the RXLR effectors in this present study to demonstrate that eight of them (SFI1–SFI8) were able to suppress early flg22-triggered patter-triggered immunity (PTI) responses. Recently, Yin *et al.* (2017) have demonstrated that some of the more highly conserved PiRXLRs (termed CREs; Supplementary Table S1) are able to suppress immunity. Here, we have identified 45 of 52 tested PiRXLR effectors which enhance *P. infestans* colonization by potentially suppressing immune signalling. It is likely that overexpressing effectors in *N. benthamiana* prior to *P. infestans* inoculation results in them being in place in sufficient quantities to suppress immunity efficiently as soon as it is activated by perception of the inoculated *P. infestans*. An illustration of this principle can be seen with effector Pi04314 which supresses PTI when transgenically expressed in potato. A Pi04314 mutant form that no longer binds its S factor target, PP1c, no longer enhances infection in an ATTA (Boevink *et al.*, 2016*b*).

### RXLR effectors that target different immune pathways provide additive enhancement of growth

Importantly, we were able to demonstrate that co-expression of PiRXLR effectors that target different immune pathways can provide an additive enhancement of colonization. In contrast, effectors targeting the same pathway or process did not provide an additive effect (Fig. 6; Supplementary Fig. S12). This provides a simple assay to determine whether effectors target distinct processes in the host, based on an additive effect of their combination, or are redundant in their activity. Critically, as many effectors are likely to be delivered together to act in concert, ways to study their combined effect are necessary to proceed beyond the current studies of single effectors in isolation. In order to understand possible common features in the molecular mechanisms underlying the modes of action of these effectors *in planta*, it would be necessary to extend the analysis of these virulence-promoting RXLR effectors to identify their plant target proteins.

In conclusion, the subcellular locations of 71 PiRXLR effectors, and whether their expression in plant cells enhances *P. infestans* colonization of host leaves, provides a broad platform of confident effector candidates from which to investigate the functional mechanisms by which these effectors, singly and in combination, contribute to causing late blight. This will open up new avenues to identify and explore novel approaches to control this disease.

## Supplementary data

Supplementary data are available at *JXB* online.

Fig. S1. Western blots demonstrating the stability of GFP fusion proteins.

Fig. S2. Confocal projection images of cytoplasmic and nucleo-cytoplasmic effectors.

Fig. S3. Confocal projection images of plasma membrane-associated effectors.

Fig. S4. Confocal projection images of nuclear-associated effectors.

Fig. S5. Confocal projection images of effectors associated with microtubules and mitochondria.

Fig. S6. GFP–Pi04049 co-expressed with subcellular markers.

Fig. S7. Further images of co-expression of CFP–Pi16663, YFP–StSec5, and the peroxisome marker.

Fig. S8. CFP–Pi16663, the peroxisome marker, and YFP–StSec5 localization around *P. infestans* haustoria.

Fig. S9. Differential recognition of Avr-blb2 family members and recognition of mis-targeted Pi04097.

Fig. S10. Intracellular Pi04314 immunoprecipitates PP1c-1 but secreted Pi04314 does not.

Fig. S11. Cell death responses triggered by expression of Pi08174 and Pi10232.

Fig. S12. Effector combination co-expression can provide an additive enhancement of colonization

Table S1. Features of the *P. infestans* RXLR effectors examined in this study and other published studies.

Table S2. Primers used in this study.

Table S3. List of synthesized effectors.

Supplementary Tables S1-S3 and Figures S1-S12
