# Supplementary material for: Phytophthora infestans RXLR effectors act in concert at diverse subcellular locations to enhance host colonization
Source: J Exp Bot. 2018 Oct 16;70(1):343–56. doi: 10.1093/jxb/ery360 (PMC6305197; doi:10.1093/jxb/ery360)
Supplement: Supplementary Tables S1-S3 and Figures S1-S12 [file ery360_suppl_supplementary_tables_s1-s3_figures_s1-s12.pdf]

Supplementary Table S1: Features of the *P. infestans* RXLR effectors examined in this study and other published studies

PITG_	Family	Other names	Studies supporting expression during infection	Localisation of GFP-effector fusion fluorescence	Accumulation at haustoria	Enhancement of *P. infestans* colonisation	Publication of infection enhancement[b] and localisation[c]
00366	RxLRfam80	PexRD43	Whisson *et al*., 2007; Yin *et al*., 2017; Ah Fong *et al.,* 2017	nucleo-cytoplasmic	N	Y	this work
00582	RxLRsng212		Haas *et al*., 2009; Cooke *et al*.,2012; Yin *et al*., 2017; Ah Fong *et al.,* 2017	plasma membrane; forms 'crystal-like' structures	nd	N	this work
00821	RxLRfam108	PexRD21, CRE1	Oh *et al*., 2009, Yin *et al*., 2017; Ah Fong *et al.,* 2017	plasma membrane	Y	Y	this work, Yin *et al*., 2017[b]
02860	RxLRfam80		Haas *et al*., 2009; Cooke *et al*., 2012; Yin *et al*., 2017; Ah Fong *et al.,* 2017	nucleo-cytoplasmic	N	Y	Yang *et al*., 2016[bc]
03192	RxLRfam66	PexRD28	Whisson *et al*., 2007; Oh *et al*., 2009; McLellan *et al*., 2013; Yin *et al*., 2017; Ah Fong *et al.,* 2017	endoplasmic reticulum	N	Y	McLellan *et al*., 2013[bc]
04049	RxLRfam67		Haas *et al*., 2009; Yin *et al*., 2017; Ah Fong *et al.,* 2017	nucleo-cytoplasmic and associated with small mobile bodies	nd	Y	this work

PITG_	Family	Other names	Studies supporting expression during infection	Localisation of GFP-effector fusion fluorescence	Accumulation at haustoria	Enhancement of *P. infestans* colonisation	Publication of infection enhancement[b] and localisation[c]
04089	RxLRfam5	PexRD41	Whisson *et al*., 2007; Haas *et al*., 2009; Oh *et al*., 2009; Cooke *et al*., 2012; Yin *et al*., 2017; Ah Fong *et al.,* 2017	nuclear; nucleolar rings; cytoplasmic background	N	Y	Wang *et al*., 2015[bc]
04097	RxLRfam5	avrblb2 family, SFI1	Haas *et al*., 2009; Cooke *et al*., 2012; Zheng *et al*., 2014; Yin *et al*., 2017	nuclear; nucleolar; cytoplasmic background	Y	Y	Zheng *et al*., 2014[bc]
04145	RxLRfam17	SFI2, PexRD29	Whisson *et al*., 2007; Zheng *et al*., 2014; Yin *et al*., 2017; Ah Fong *et al.,*2017	strongly nuclear; occasionally nucleolar, high expression causes cell death	N	-	Zheng *et al*., 2014[bc]
04314	RxLRfam49	PexRD24	Whisson *et al*., 2007; Oh *et al*., 2009; Cooke *et al*., 2012; Yin *et al*., 2017; Ah Fong *et al.,*2017	nuclear; nucleolar; cytoplasmic background	N	Y	Boevink *et al*., 2016b[bc]
04339	RxLRfam81	PexRD20	Whisson *et al*., 2007; Cooke *et al*., 2012; Yin *et al*., 2017; Ah Fong *et al.,* 2017	nuclear; nucleolar; cytoplasmic background	nd	Y	this work
04388	RxLRfam1	PexRD25	Whisson *et al*., 2007; Yin *et al*., 2017; Ah Fong *et al.,* 2017	nuclear; nucleolar; faintly plasma	Y	Y	this work

PITG_	Family	Other names	Studies supporting expression during infection	Localisation of GFP-effector fusion fluorescence	Accumulation at haustoria	Enhancement of *P. infestans* colonisation	Publication of infection enhancement[b] and localisation[c]
				membrane			
05750	RxLRfam29	PexRD49, CRE3	Whisson *et al*., 2007; Haas *et al*., 2009; Oh *et al*., 2009; Cooke *et al*., 2012, Yin *et al*., 2017; Ah Fong *et al.*,2017	plasma membrane; nuclear/nucleolar	Y	Y	this work, Yin *et al*., 2017[b]
05911/ 05912/ 22089	RxLRfam18		Cooke *et al*., 2012; Yin *et al*., 2017; Ah Fong *et al.,* 2017	nucleo-cytoplasmic	nd	Y	this work
06087	RxLRfam87	PexRD16, SFI3	Whisson *et al*., 2007; Haas *et al*., 2009; Oh *et al*., 2009; Cooke *et al*., 2012; Zheng *et al*., 2014; Yin *et al*., 2017; Ah Fong *et al.,* 2017	nuclear; nucleolar rings; cytoplasmic background	N	Y	Zheng *et al*., 2014[bc]
06094	RxLRfam36		Cooke *et al*., 2012; Yin *et al*., 2017; Ah Fong *et al.,* 2017	nuclear; nucleolar rings; cytoplasmic background	nd	Y	this work
06099	RxLRfam36	PexRD50	Whisson *et al*., 2007; Haas *et al*., 2009; Oh *et al*., 2009; Cooke *et al*., 2012; Yin *et al*., 2017	nucleo-cytoplasmic	N	Y	this work
06308	RxLRfam23	CRE5	Haas *et al*., 2009; Yin *et al*., 2017; Yin *et al*., 2017; Ah Fong *et al.,* 2017	nuclear; nucleolar (decreasing with time); cytoplasmic	N	Y	this work, Yin *et al*., 2017[b]

PITG_	Family	Other names	Studies supporting expression during infection	Localisation of GFP-effector fusion fluorescence	Accumulation at haustoria	Enhancement of *P. infestans* colonisation	Publication of infection enhancement[b] and localisation[c]
				background			
06478	RxLRfam16	PexRD18	Whisson *et al*., 2007; Haas *et al*., 2009; Cooke *et al*., 2012; Yin *et al*., 2017; Ah Fong *et al.,* 2017	plasma membrane; cytoplasmic background	N	Y	this work
07387	RxLRfam52	Avr4	Haas *et al*., 2009; Cooke *et al*., 2012; Yin *et al*., 2017	nuclear; nucleolar; sub-nuclear bodies; cytoplasm; microtubules	nd	Y	this work
07550	RxLRfam117	AvrSmira 1	Cooke *et al*., 2012; Yin *et al*., 2017; Ah Fong *et al.,* 2017	NA (GFP-fusion is unstable)	-		
07550 ED				nuclear; nucleolar	nd	Y	this work
07555	RxLRsng247		Haas *et al*., 2009; Cooke *et al*., 2012; Yin *et al*., 2017; Ah Fong *et al.,* 2017	nucleo-cytoplasmic	nd	Y	this work
08174	RxLRfam19		Haas *et al*., 2009; Yin *et al*., 2017; Ah Fong *et al.,* 2017	cytoplasmic; causes cell death	nd	-	this work
08278	RxLRfam7		Haas *et al*., 2009; Ah Fong *et al.,* 2017	nucleo-cytoplasmic	nd	N	this work
08943 / 22870	RxLRfam7	Avr2	Haas *et al*., 2009; Cooke *et al*., 2012; Yin	plasma membrane	Y[a]	Y	Saunders *et al*. 2012[bc], Turnbull *et*

PITG_	Family	Other names	Studies supporting expression during infection	Localisation of GFP-effector fusion fluorescence	Accumulation at haustoria	Enhancement of *P. infestans* colonisation	Publication of infection enhancement[b] and localisation[c]
ED			*et al*., 2017; Ah Fong *et al.,* 2017				*al*., 2017[b]
09218	RxLRfam55		Haas *et al*., 2009; Yin *et al*., 2017; Ah Fong *et al.,* 2017	mitochondria; faintly endoplasmic reticulum	nd	Y	this work
09316	RxLRfam16	RD54	Cooke *et al*., 2012; Yin *et al*., 2017; Ah Fong *et al.,* 2017	autophagosomes; nucleo-cytoplasmic	nd	Y	Dagdas *et al*., 2016[bc]
09585	RxLRfam90	SFI4	Cooke *et al*., 2012; Zheng *et al*., 2014; Yin *et al*., 2017; Ah Fong *et al.,* 2017	nucleo-cytoplasmic	nd	Y	Zheng *et al*., 2014[bc]
09732	RxLRfam1		Haas *et al*., 2009; Cooke *et al*., 2012; Yin *et al*., 2017; Ah Fong *et al.,* 2017	NA (GFP-fusion is unstable)	-		
09732 ED				plasma membrane; faint nucleolar; cytoskeleton-like strands	nd	Y	this work
10232	RxLRfam69		Cooke *et al*., 2012; Yin *et al*., 2017; Ah Fong *et al.,* 2017	nucleo-cytoplasmic; causes cell death	nd	-	this work
10654	RxLRfam46		Haas *et al*., 2009; Cooke *et al*., 2012; Yin *et al*., 2017; Ah Fong *et*	nucleo-cytoplasmic	nd	Y	this work

PITG_	Family	Other names	Studies supporting expression during infection	Localisation of GFP-effector fusion fluorescence	Accumulation at haustoria	Enhancement of *P. infestans* colonisation	Publication of infection enhancement[b] and localisation[c]
			al., 2017				
11383	RxLRfam6	PexRD2	Whisson *et al*., 2007; Oh *et al*., 2009; King *et al*., 2014	nucleo-cytoplasmic	N	Y	King *et al*., 2014[bc]
11484	RxLRfam120	Avr10	Haas *et al*., 2009; Yin *et al*., 2017; Ah Fong *et al.,* 2017	nucleo-cytoplasmic	N	Y	this work
11507	RxLRfam120		Haas *et al*., 2009	nucleo-cytoplasmic	nd	Y	this work
12731	RxLRfam1		Cooke *et al*., 2012; Yin *et al*., 2017; Ah Fong *et al.,* 2017	nucleo-cytoplasmic	nd	Y	this work
12737	RxLRfam43		Haas *et al*., 2009; Cooke *et al*., 2012; Yin *et al*., 2017; Ah Fong *et al.,* 2017	nucleo-cytoplasmic	nd	Y	this work
13093	RxLRfam38		Haas *et al*., 2009; Cooke *et al*., 2012; Ah Fong *et al.,* 2017	nucleo-cytoplasmic	nd	Y	this work
13625	not on list			nucleo-cytoplasmic	nd	Y	this work
13628	RxLRfam6	SFI5, PexRD27	Whisson *et al*., 2007; Oh *et al*., 2009; Cooke *et al*., 2012; Zheng *et al*., 2014; Yin *et al*., 2017; Ah Fong *et al.,*	plasma membrane; faint nucleolar	N	Y	Zheng *et al*., 2014[bc]; Zheng *et al*., 2018[c]

PITG_	Family	Other names	Studies supporting expression during infection	Localisation of GFP-effector fusion fluorescence	Accumulation at haustoria	Enhancement of *P. infestans* colonisation	Publication of infection enhancement[b] and localisation[c]
			2017				
13959	RxLRfam3	SFI6	Zheng *et al*., 2014; Yin *et al*., 2017	nucleo-cytoplasmic	N	Y	Zheng *et al*., 2014[bc]
14371	RxLRfam58	Avr3a, PexRD7	Whisson *et al*., 2007; Haas *et al*., 2009; Oh *et al*., 2009; Cooke *et al*., 2012; Yin *et al*., 2017; Ah Fong *et al.,* 2017	nucleo-cytoplasmic	N	N	Bos *et al*., 2010[bc]
14783	RxLRfam6		Haas *et al*., 2009; Yin *et al*., 2017; Ah Fong *et al.,* 2017	nucleo-cytoplasmic	nd	Y	this work
14788	RxLRfam8		Haas *et al*., 2009; Yin *et al*., 2017	microtubule-associated; some nucleolar and cytoplasmic	nd	Y	this work
15110	RxLRfam1		Haas *et al*., 2009; Cooke *et al*., 2012; Yin *et al*., 2017; Ah Fong *et al.,* 2017	microtubule-associated	nd	Y	this work
15127	RxLRfam1		Cooke *et al*., 2012; Yin *et al*., 2017	nucleo-cytoplasmic and plasma membrane	nd	Y	this work
15278	RxLRfam1		Cooke *et al*., 2012; Yin *et al*., 2017; Ah Fong *et al.,* 2017	cytoplasmic	nd	Y	this work

PITG_	Family	Other names	Studies supporting expression during infection	Localisation of GFP-effector fusion fluorescence	Accumulation at haustoria	Enhancement of *P. infestans* colonisation	Publication of infection enhancement[b] and localisation[c]
15287	RxLRfam96	PexRD1	Whisson *et al*., 2007; Oh *et al*., 2009; Yin *et al*., 2017	variable but often nuclear-associated	nd	Y	this work
15679	RxLRfam23		Haas *et al*., 2009; Yin *et al*., 2017	nucleo-cytoplasmic	nd	Y	this work
15972	RxLRfam7		Haas *et al*., 2009	nucleo-cytoplasmic	nd	Y	this work
16195	RxLRfam1		Haas *et al*., 2009; Cooke *et al*., 2012; Yin *et al*., 2017; Ah Fong *et al.,* 2017	plasma membrane	nd	Y	this work
16427/ 16240/ 16233,	RxLRfam9	PexRD12 , CRE13	Whisson *et al*., 2007; Oh *et al*., 2009; Cooke *et al*., 2012, Yin *et al*., 2017; Ah Fong *et al.,* 2017	variable, often nucleo-cytoplasmic but sometimes plasma membrane; occasional faint nucleolar rings	Y	N	this work
16294	RxLRfam97	Avrpi-vnt1	Haas *et al*., 2009; Cooke *et al*., 2012; Yin *et al*., 2017; Ah Fong *et al.,* 2017	nuclear; nucleolar; cytoplasmic background	nd	N	this work
16663	RxLRfam2	Avr1	Haas *et al*., 2009; Cooke *et al*., 2012; Yin *et al*., 2017	nucleo-cytoplasmic; later peroxisomes	Y	Y	Du *et al*., 2015a,b[bc]; this work

PITG_	Family	Other names	Studies supporting expression during infection	Localisation of GFP-effector fusion fluorescence	Accumulation at haustoria	Enhancement of *P. infestans* colonisation	Publication of infection enhancement[b] and localisation[c]
16705	RxLRfam1		Haas *et al*., 2009; Yin *et al*., 2017; Ah Fong *et al.,* 2017	cytoplasmic	nd	Y	this work
16737	RxLRfam8	PexRD15	Whisson *et al*., 2007; Yin *et al*., 2017; Ah Fong *et al.,* 2017	plasma membrane; faint nucleolar; occasional sub-nuclear bodies	N	Y	this work
17063	RxLRfam45	PexRD44, CRE14	Haas *et al*., 2009; Oh *et al*., 2009; Yin *et al*., 2017; Ah Fong *et al.,* 2017	plasma membrane; nucleolar	N	Y	this work, Yin *et al*., 2017[b]
17316/ 17309	RxLRfam1	CRE15	Cooke *et al*., 2012; Yin *et al*., 2017; Ah Fong *et al.,* 2017	plasma membrane; faint nucleolar	Y	Y	this work; Murphy et al 2018[bc]
18215	RxLRfam124	Avr3b, SFI7	Haas *et al*., 2009; Cooke *et al*., 2012; Zheng *et al*., 2014; Yin *et al*., 2017	variably nucleo-cytoplasmic or plasma membrane associated	N	Y	Zheng *et al*., 2014[bc]; this work
18609	RxLRfam26		Haas *et al*., 2009; Cooke *et al*., 2012; Yin *et al*., 2017; Ah Fong *et al.,* 2017	nuclear; nucleolar; cytoplasmic background	nd	Y	this work
18670	RxLRfam5		Haas *et al*., 2009; Yin *et al*., 2017; Ah Fong *et*	nuclear, nucleolar,	nd	Y	this work

PITG_	Family	Other names	Studies supporting expression during infection	Localisation of GFP-effector fusion fluorescence	Accumulation at haustoria	Enhancement of *P. infestans* colonisation	Publication of infection enhancement[b] and localisation[c]
			al., 2017	cytoplasmic background			
19617	RxLRfam7		Haas *et al*., 2009; Yin *et al*., 2017	nucleo-cytoplasmic	nd	Y	this work
20300	RxLRfam5	avrblb2 family, PexRD39/40	Whisson *et al*., 2007; Cooke *et al*., 2012; Yin *et al*., 2017; Ah Fong *et al.,* 2017	plasma membrane and rarely, faint nucleolar	Y[a]	Y	Bozkurt *et al.,* 2011[bc]; this work
20303	RxLRfam5	avrblb2 family, SFI8	Haas *et al*., 2009; Cooke *et al*., 2012; Zheng *et al*., 2014; Ah Fong *et al.,* 2017	nucleo-cytoplasmic	nd	Y	Zheng *et al*., 2014[bc]
21388	RxLRfam54	avrblb1 family, IPIO1, PexRD6	Whisson *et al*., 2007; Haas *et al*., 2009; Oh *et al*., 2009; Cooke *et al*., 2012; Yin *et al*., 2017; Ah Fong *et al.,* 2017	nuclear; strongly nucleolar	nd	N	this work
IPIO4 (not in reference strain)	RxLRfam54	avrblb1 family, IPIO4	Champouret *et al*., 2009	plasma membrane; occasional nucleolar	nd	nd	this work
22724	RxLRfam67		Haas *et al*., 2009; Yin *et al*., 2017; Ah Fong *et al.,* 2017	nucleo-cytoplasmic	nd	Y	this work
22757	RxLRsng203		Haas *et al*., 2009; Yin *et*	nuclear;	nd	Y	this work

PITG_	Family	Other names	Studies supporting expression during infection	Localisation of GFP-effector fusion fluorescence	Accumulation at haustoria	Enhancement of *P. infestans* colonisation	Publication of infection enhancement[b] and localisation[c]
			al., 2017; Ah Fong *et al.,* 2017	nucleolar; sub-nuclear bodies; cytoplasmic background			
22798	RxLRsng157		Haas *et al*., 2009; Yin *et al*., 2017	nuclear; nucleolar; sub-nuclear bodies; cytoplasmic background	nd	Y	Wang *et al*., 2017[bc]
22922	RxLRfam2		Cooke *et al*., 2012; Yin *et al*., 2017; Ah Fong *et al.,* 2017	variable but often plasma membrane and faintly nucleolar	nd	Y	this work
22926/ 05910	RxLRfam52	CRE4	Cooke *et al*., 2012; Yin *et al*., 2017; Ah Fong *et al.,* 2017	nuclear; nucleolar; cytoplasmic background	nd	Y	this work and Wang *et al*., 2018b
23015	RxLRfam100	CRE16	Haas *et al*., 2009; Yin *et al*., 2017; Ah Fong *et al.,* 2017	nuclear; nucleolar; cytoplasmic background; low fluorescence	nd	Y	this work
23226	RxLRfam100	CRE18	Haas *et al*., 2009; Yin *et al*., 2017; Ah Fong *et al.,* 2017	nucleo-cytoplasmic; occasionally nucleolar; low	nd	Y	this work

**PITG_**	**Family**	**Other names**	**Studies supporting expression during infection**	**Localisation of GFP-effector fusion fluorescence**	**Accumulation at haustoria**	**Enhancement of *P. infestans* colonisation**	**Publication of infection enhancement[b] and localisation[c]**
				fluorescence			

[a] Published by others

nd = not determined

Supplementary Table S2 Primers used in this study

Primer Name	Sequence	Target
attB-F2	GGGGACAAGTTTGTACAAAAAAGCAGGCT	For nested PCR
attB-R2	GGGGACCACTTTGTACAAGAAAGCTGGG	For nested PCR
04314gatewayF1	AAAGCAGGCTTCACCATGGTATCGACCGAAGCTAAT	Pi04314/SP-Pi04314
04314gatewayR1	GAAAGCTGGGTC TCACGAGTTGGTTTTGTA	Pi04314/SP-Pi04314
22926gatewayF1	AAAGCAGGCTTCACCATGAAGCCTCTCCCGTTAGCA	Pi22926/SP-Pi22926
22926gatewayR1	GAAAGCTGGGTCTCATGTGGTAAGCTTCGT	Pi22926/SP-Pi22926
10654Bam5	CTGGATCCACTATGACTCCCACTGACTCTCAGCA	Pi10654
3Xho10654st	ATCTCGAGTTAGATCCATTCTTCAGGGAT	Pi10654
5Bam07550dR	AGGATCCATAAGATTCAGACAAGCTTAATC	Pi07550
3Xho07550	ACTCGAGTTATCCGGAGGGGTTTAGCGAGT	Pi07550
5Sal09732dR	TTAGTCGACGGCATCGGGGGCACCGCGAT	Pi09732
3EcoRV09732	CGATATCTCAGTTTCCCGGCTTTTGACTC	Pi09732
5StSec5attB1	GGGGACAAGTTTGTACAAAAAAGCAGGCTCAATAATGTCAACCGACACTGACGACGAA	StSec5
3StSec5attB2	GGGGACCACTTTGTACAAGAAAGCTGGGGTTTATCTACGTCTGGGC	StSec5
00366_F	AAAGCAGGCTTCACCATGAACGTGCTACATGTACCG	Pi00366
00366_R	GAAAGCTGGGTCCTAAGTATGAAATGCTGAAG	Pi00366
00821_F	AAAGCAGGCTTCACCATGACCCCCGTCATAAAAGAA	Pi00821
00821_R	GAAAGCTGGGTCTTATCTCCAGCTTCTGCC	Pi00821
04090_F	AAAAAGCAGGCTCACAAATGTTCCCAATCCCCGACGAGTCTCG	Pi04090
04090_R	AGAAAGCTGGGTCACCGCCTCCGGATCAGGACTTCGTCATTTTTGCTTTTGC	Pi04090
04339_F	AAAGCAGGCTTCACCATGACGACGGACGCCCAGCTGA	Pi04339
04339_R	GAAAGCTGGGTCCTATACTTCCACGGGCAGGGG	Pi04339
04388_F	AAAGCAGGCTTCACCATGGAGCAAGCTGCCGCAGCC	Pi04388
04388_R	GAAAGCTGGGTCTTACAGCTGGTGCTTCTTG	Pi04388
05750_F	AAAGCAGGCTTCACCATGCGCTCGGCCACCGAACATG	Pi05750
05750_R	GAAAGCTGGGTCTTACGTAGCAGCAGTCTTG	Pi05750

Primer Name	Sequence	Target
05911_F	AAAGCAGGCTTCACCATGGCTACGAGCTCCGATCAG	Pi05911
05911_R	GAAAGCTGGGTCTCAACTATTTATCCTGTTC	Pi05911
06094_F	AAAGCAGGCTTCACCATGTCCGACTCGGAGAAAATC	Pi06094
06094_R	GAAAGCTGGGTCCTAATACTCCTTAGCCTTG	Pi06094
06099_F	AAAGCAGGCTTCACCATGTCGGACTCGGAGAAAGCTG	Pi06099
06099_R	GAAAGCTGGGTCCTAAAAGTCTTCTGCCTTGCAAAA	Pi06099
06308_F	AAAAAGCAGGCTCACAAATGTCGATCTCTTCTTTCTCCG	Pi06308
06308_R	AGAAAGCTGGGTCACCGCCTCCGGAGGAAAGAGCACGACATACTCCTCC	Pi06308
06478_F	AAAGCAGGCTTCACCATGCAAACACCCCCTGGACAAGC	Pi06478
06478_R	GAAAGCTGGGTCTCACTGAGCTTTCATAGCTG	Pi06478
07387_F	AAAGCAGGCTTCACCATGGATTCTTTAGCTCGTACC	Pi07387
07387_R	GAAAGCTGGGTCCTAAGATATGGGCCGTCT	Pi07387
07550_F	AAAGCAGGCTTCACCATGGCAACTGTGACCAAGGTA	Pi07550
07550_R	GAAAGCTGGGTCTTATCCGGAGGGGTTTAG	Pi07550
08278_F	AAAAAGCAGGCTCACAAATGGCTCTCCCTTCGGCTAAGG	Pi08278
08278_R	AGAAAGCTGGGTCACCGCCTCCGGATTAGTCCTTTCCGATCGTTTCC	Pi08278
09732_F	AAAGCAGGCTTCACCATGACCCCCTCGGAGCTCACC	Pi09732
09732_R	GAAAGCTGGGTCTCAGTTTCCCGGCTTTTGAC	Pi09732
12731_F	AAAGCAGGCTTCACCATGTCAGGCATCCAGGCTATTG	Pi12731
12731_R	GAAAGCTGGGTCTCATCCTTCTTTCTTCATC	Pi12731
13625_F	AAAGCAGGCTTCACCATGAAAGAACTCACAAACATC	Pi13625
13625_R	GAAAGCTGGGTCTCACGACAACACATTCCTTA	Pi13625
14783_F	AAAGCAGGCTTCACCATGCTCGTGAACTCGAACCAA	Pi14783
14783_R	GAAAGCTGGGTCTTATAAATGGTAGAAAGTAGG	Pi14783
15278_F	AAAGCAGGCTTCACCATGGGTGTGACCTTCAATGAG	Pi15278
15278_R	GAAAGCTGGGTCTCACACTTTGGCAAATGCATT	Pi15278
15287_F	AAAGCAGGCTTCACCATGCTATCTGCCCATCGGGCGC	Pi15287
15287_R	GAAAGCTGGGTCTTATTTAAAAACTACTTTTGAGTATTC	Pi15287

Primer Name	Sequence	Target
15679_F	AAAAAGCAGGCTCACAAATGGACCCAAGCGGTCTTGCG	Pi15679
15679_R	AGAAAGCTGGGTCACCGCCTCCGGATCAACGTACTCCTCCTTCTTCG	Pi15679
16195_F	AAAGCAGGCTTCACCATGGCTTCGACGTCCAAGCTG	Pi16195
16195_R	GAAAGCTGGGTCTTATCCTGCTATATAAATCCC	Pi16195
16427_F	AAAGCAGGCTTCACCATGTTGACCACGACTGTGGCTG	Pi16427
16427_R	GAAAGCTGGGTCTTACTGGTTCTTCCACCAC	Pi16427
16737_F	AAAGCAGGCTTCACCATGACAAGGGAATTGAATATGAGG	Pi16737
16737_R	GAAAGCTGGGTCTCAATATTTCATCACTGTGG	Pi16737
51_2006F	AAAGCAGGCTTCACCATGTTCGACCACGACAAGGT	Pi16663
51_2006R	GAAAGCTGGGTCTTAAAATGGTACCACAACATG	Pi16663
17063_F	AAAGCAGGCTTCACCATGGCTTCGGCTGATTCGAATGAGC	Pi17063
17063_R	GAAAGCTGGGTCTTACTTTGCAAGATGGGAGC	Pi17063
17316_F	AAAGCAGGCTTCACCATGGCTGATGCCAAGGCTGGA	Pi17316
17316_R	GAAAGCTGGGTCTCATTTGCCAGACAAACGC	Pi17316
18609_F	AAAGCAGGCTTCACCATGGCCGGTGGCTCCAACCTC	Pi18609
18609_R	GAAAGCTGGGTCTCACTGCAGTTGCTGCTGAGA	Pi18609
18670_F	AAAAAGCAGGCTCACAAATGTTCCCGAATCCCGACGAAACTCG	Pi18670
18670_R	AGAAAGCTGGGTCACCGCCTCCGGACTACTTCGCTGCCTTCTTACCG	Pi18670
19617_F	AAAAAGCAGGCTCACAAATGCTCCCTTCGACTAAGGACTTG	Pi19617
19617_R	AGAAAGCTGGGTCACCGCCTCCGGATTAGTCCTTTCCGATCGTTTCC	Pi19617
21388_F	AAAGCAGGCTTCACCATGGTTTCATCCAATCTC	Pi21388
21388_R	GAAAGCTGGGTCCTAGCTAGGGCCAACGTT	Pi21388
22922_F	AAAGCAGGCTTCACCATGAAGGTATCGACGGCAGCG	Pi22922
22922_R	GAAAGCTGGGTCTCAGTGCGTGTGCTGTAG	Pi22922
22926-SP_F	AAAGCAGGCTTCACCATGAAGCCTCTCCCGTTAGCA	Pi22926
22926_R	GAAAGCTGGGTCTCATGTGGTAAGCTTCGT	Pi22926

Red text indicates *attB* sites

Supplementary Table S3. List of synthesised effectors

Pi00582
Pi04049
Pi04085
Pi07555
Pi08174
Pi09218
Pi10232
Pi10654
Pi11484
Pi11507
Pi12737
Pi13093
Pi14788
Pi15110
Pi15127
Pi15972
Pi16294
Pi16705
Pi22724
Pi22757
Pi23015
Pi23226

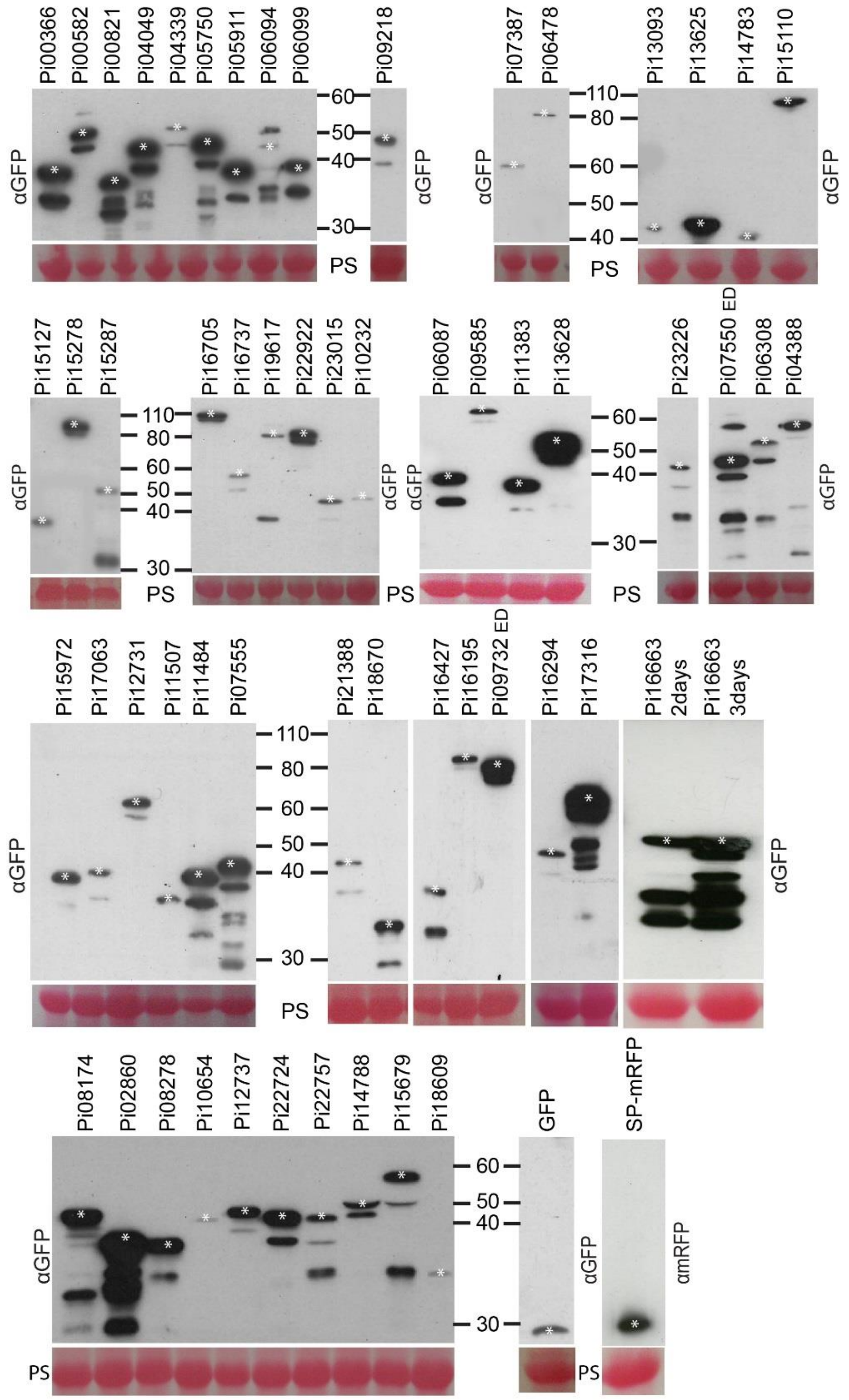

Fig.S1 The stability of N-terminally tagged RXLR effector fusion proteins. Western blots probed with mouse anti-GFP antibodies, showing all of the tested RXLR effector fusions are largely intact. The CFP-Pi16663 was examined at both 2 and 3 dpi. Blots of GFP and secreted mRFP (SP-mRFP) alone are also shown; the latter probed with an anti-mRFP antibody. Intact fusion proteins are indicated by white asterisks, size markers are indicated in kDa, and protein loading is represented by Ponceau stain (PS).

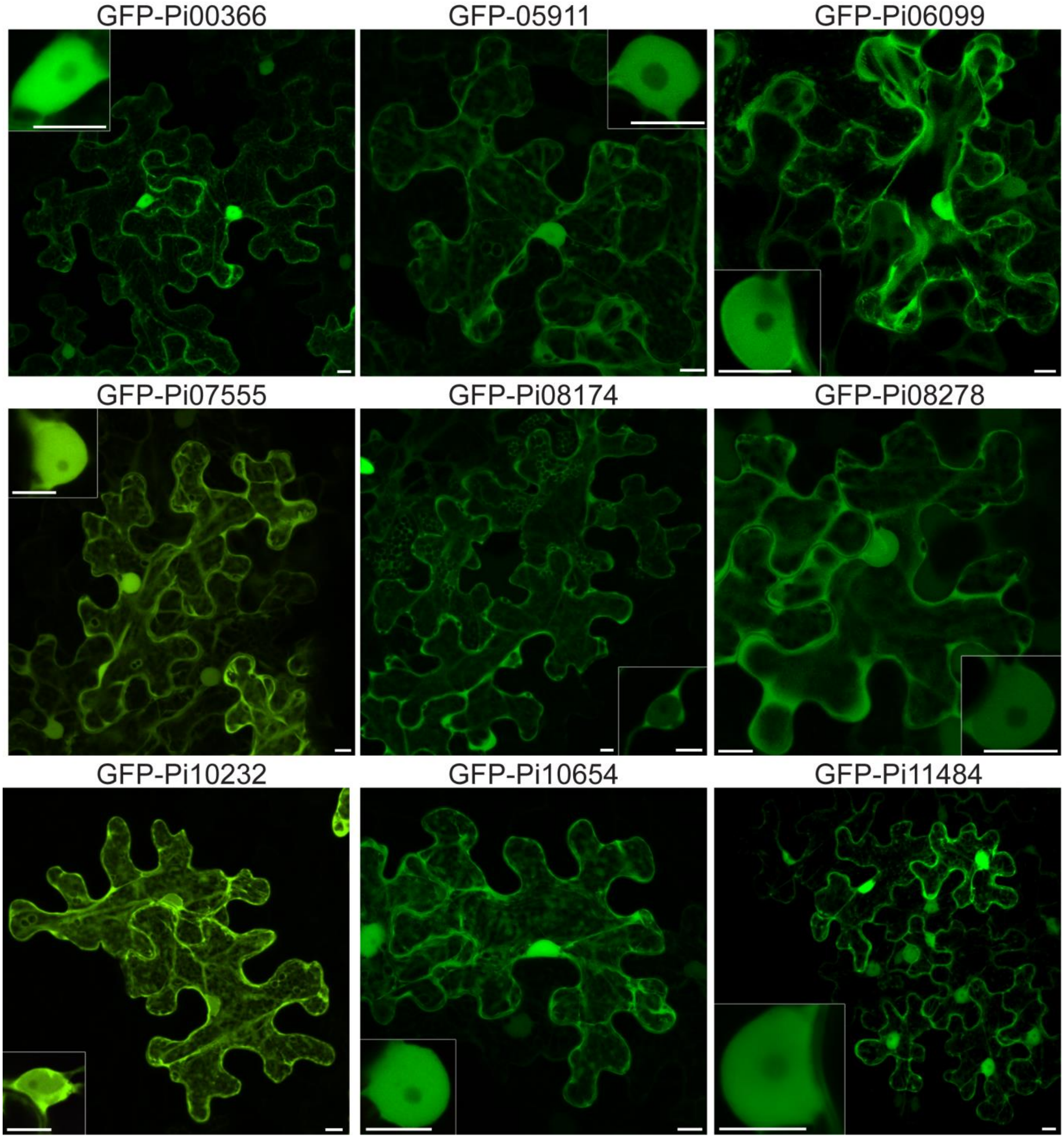
GFP-Pi00366
GFP-05911
GFP-Pi06099
GFP-Pi07555
GFP-Pi08174
GFP-Pi08278
GFP-Pi10232
GFP-Pi10654
GFP-Pi11484

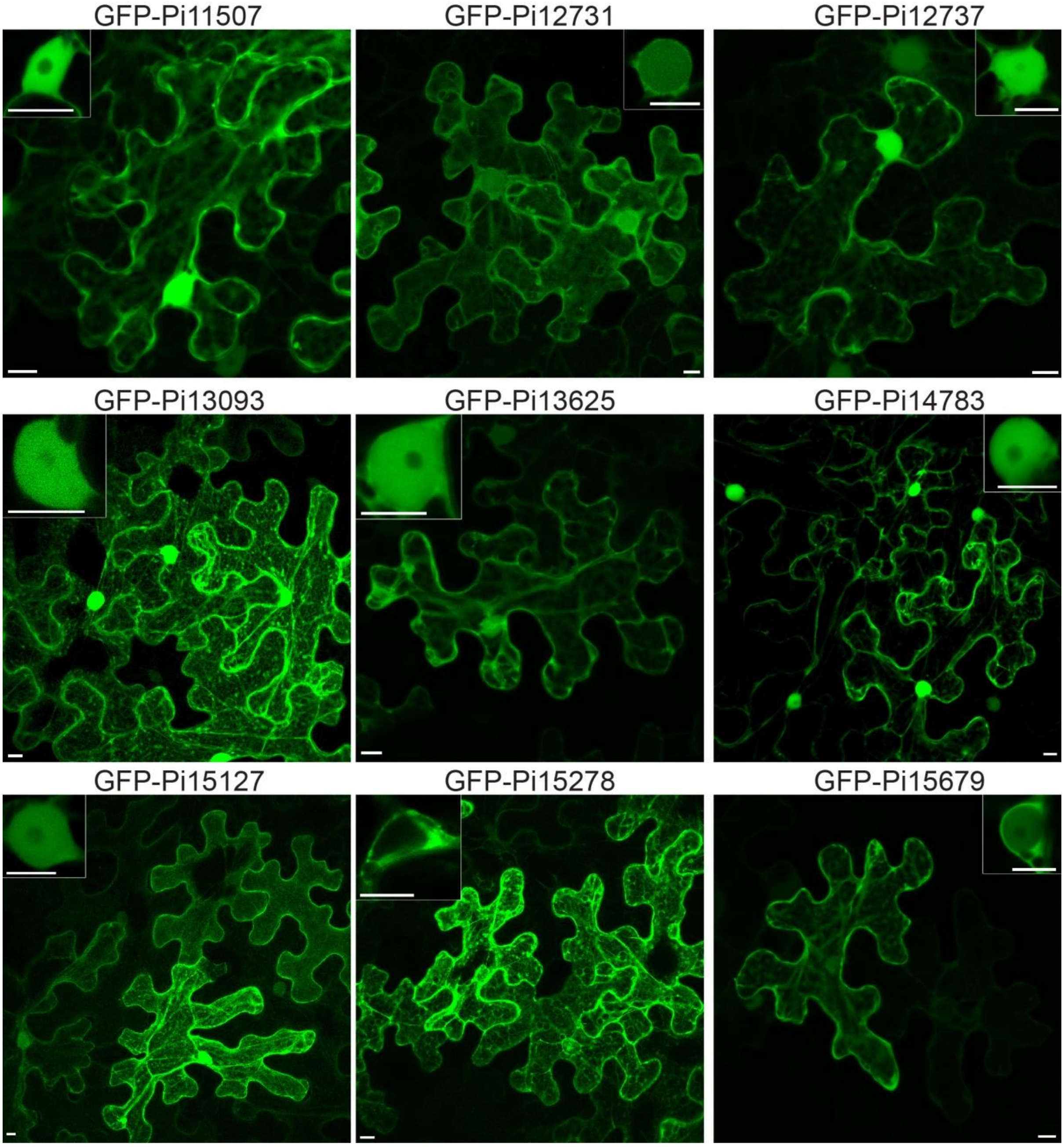
GFP-Pi11507
GFP-Pi12731
GFP-Pi12737
GFP-Pi13093
GFP-Pi13625
GFP-Pi14783
GFP-Pi15127
GFP-Pi15278
GFP-Pi15679

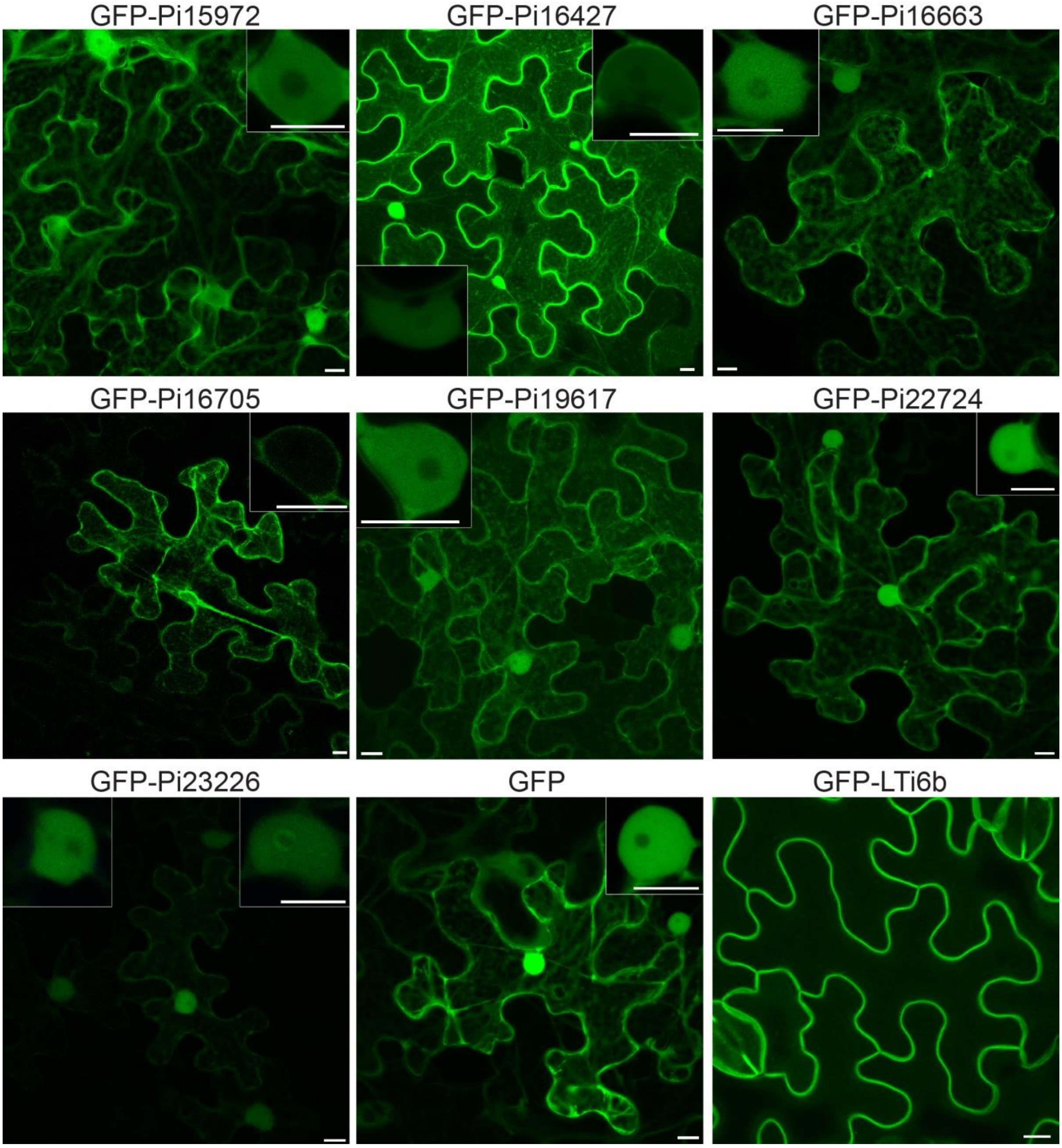

Figure S2 Nucleo-cytoplasmic and Cytoplasmic effectors

Representative confocal projection images of cells expressing GFP-effector fusions that show a dominant pattern of cytoplasmic fluorescence, most of which are nucleo-cytoplasmic. Representative images of nucleo-cytoplasmic un-fused GFP and PM-targeted GFP-LTi6b are included for comparison. Inset images are single optical sections from nuclei, most of which show no labelling of nucleoli. Second insets for GFP-Pi16427 and GFP-Pi23226 indicate that on rare occasions nucleoli appear to have rings of fluorescence around them. Scale bars represent 10 µm.

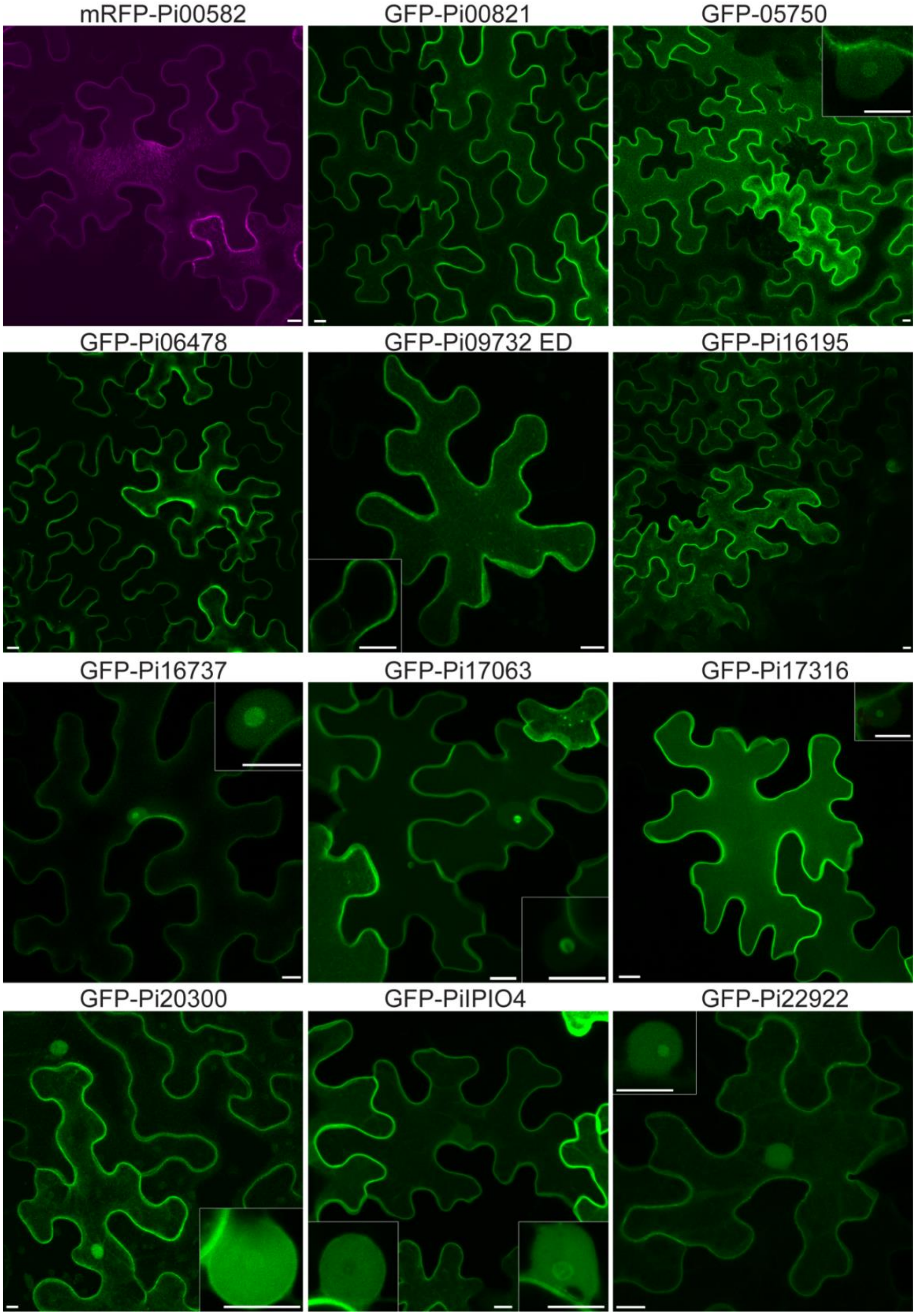

Figure S3 Plasma membrane-associated effectors

Representative confocal projection images of cells expressing FP-effector fusions that show a predominantly plasma membrane associated pattern of fluorescence. Inset images are single optical sections from nuclei of cells that also show labelling of the nucleoli. Scale bars represent 10 µm.

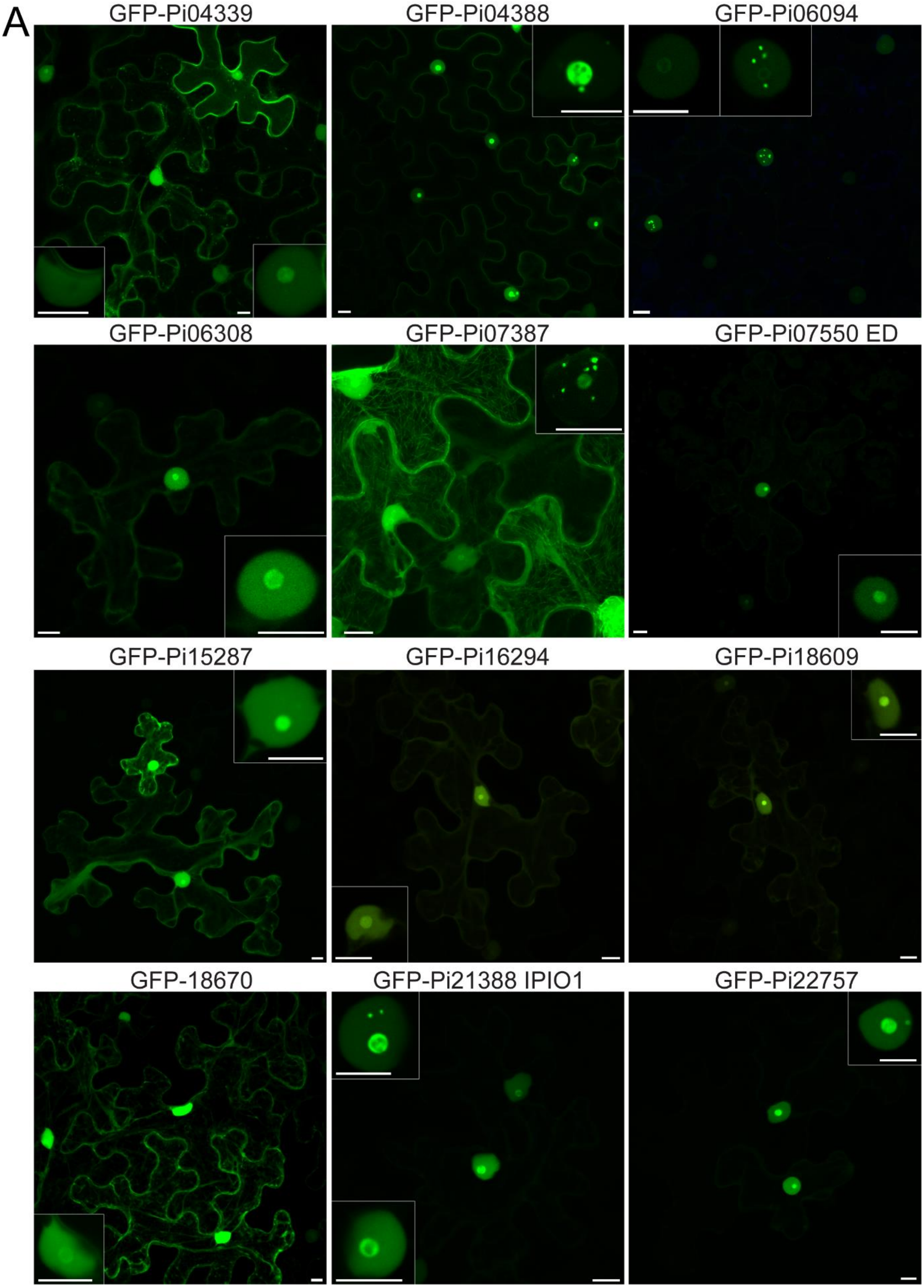
A
GFP-Pi04339
GFP-Pi04388
GFP-Pi06094
GFP-Pi06308
GFP-Pi07387
GFP-Pi07550 ED
GFP-Pi15287
GFP-Pi16294
GFP-Pi18609
GFP-18670
GFP-Pi21388 IPIO1
GFP-Pi22757

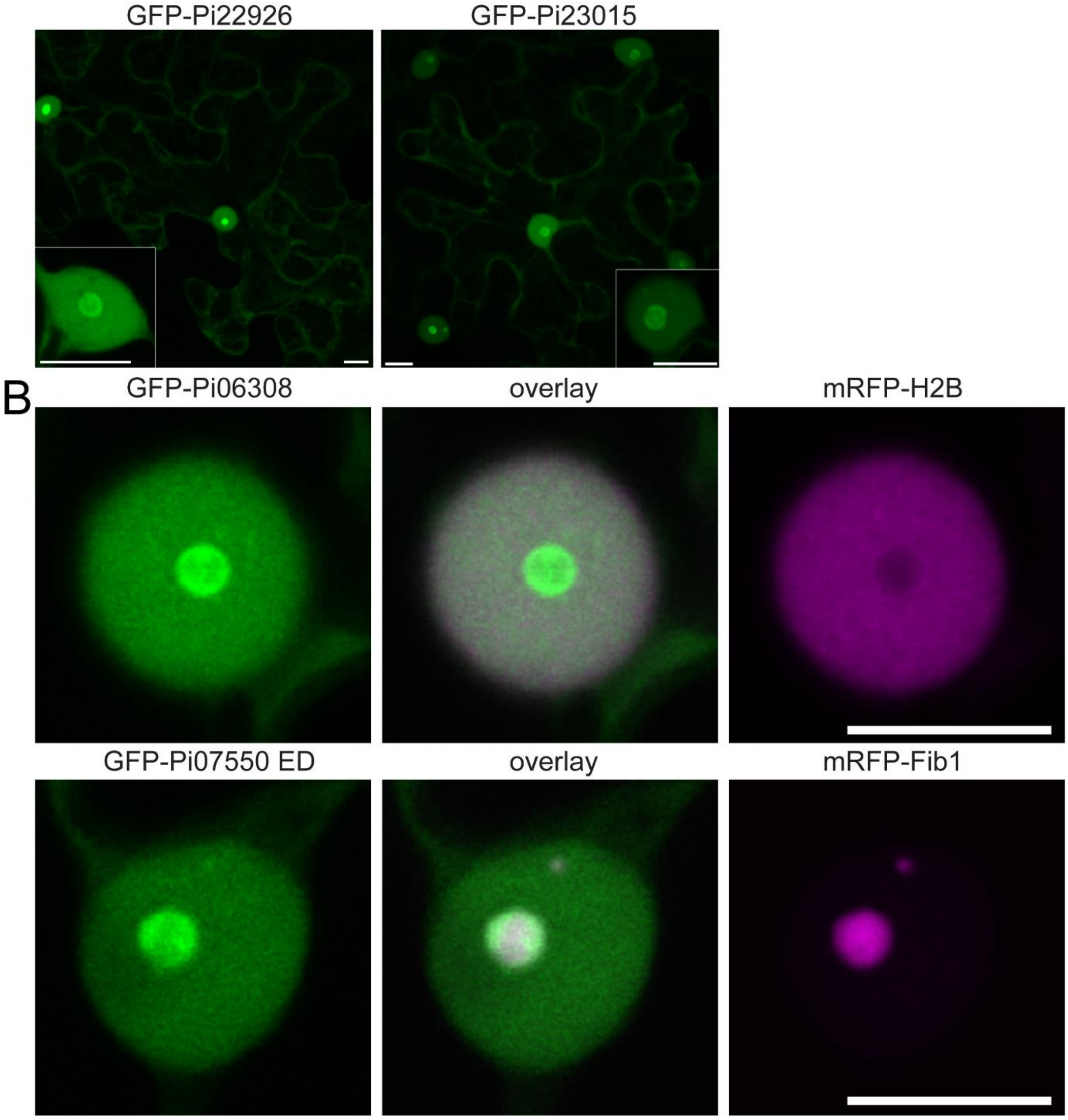

Figure S4 Nuclear-associated effectors

Representative confocal projection images of cells expressing GFP-effector fusions that show a predominantly nuclear-associated pattern of fluorescence (A). Inset images are single optical sections from nuclei. The two insets for Pi04339 are provided to indicate that an occasional nucleus did not show nucleolar labelling. The two insets for Pi06094 indicate that not all nuclei contained fluorescent sub-nuclear bodies in addition to the nucleolar fluorescence. The two insets for Pi21388 indicate that some nuclei contained fluorescent sub-nuclear bodies. Example colocalisation images of nuclear-associated effectors co-expressed with the nucleoplasm marker, mRFP tagged *N. benthamiana* histone 2B (mRFP-H2B), and the nucleolar marker mRFP-tagged *A. thaliana* Fibrillarin1 (mRFP-Fib1) (B).-Scale bars represent 10 µm.

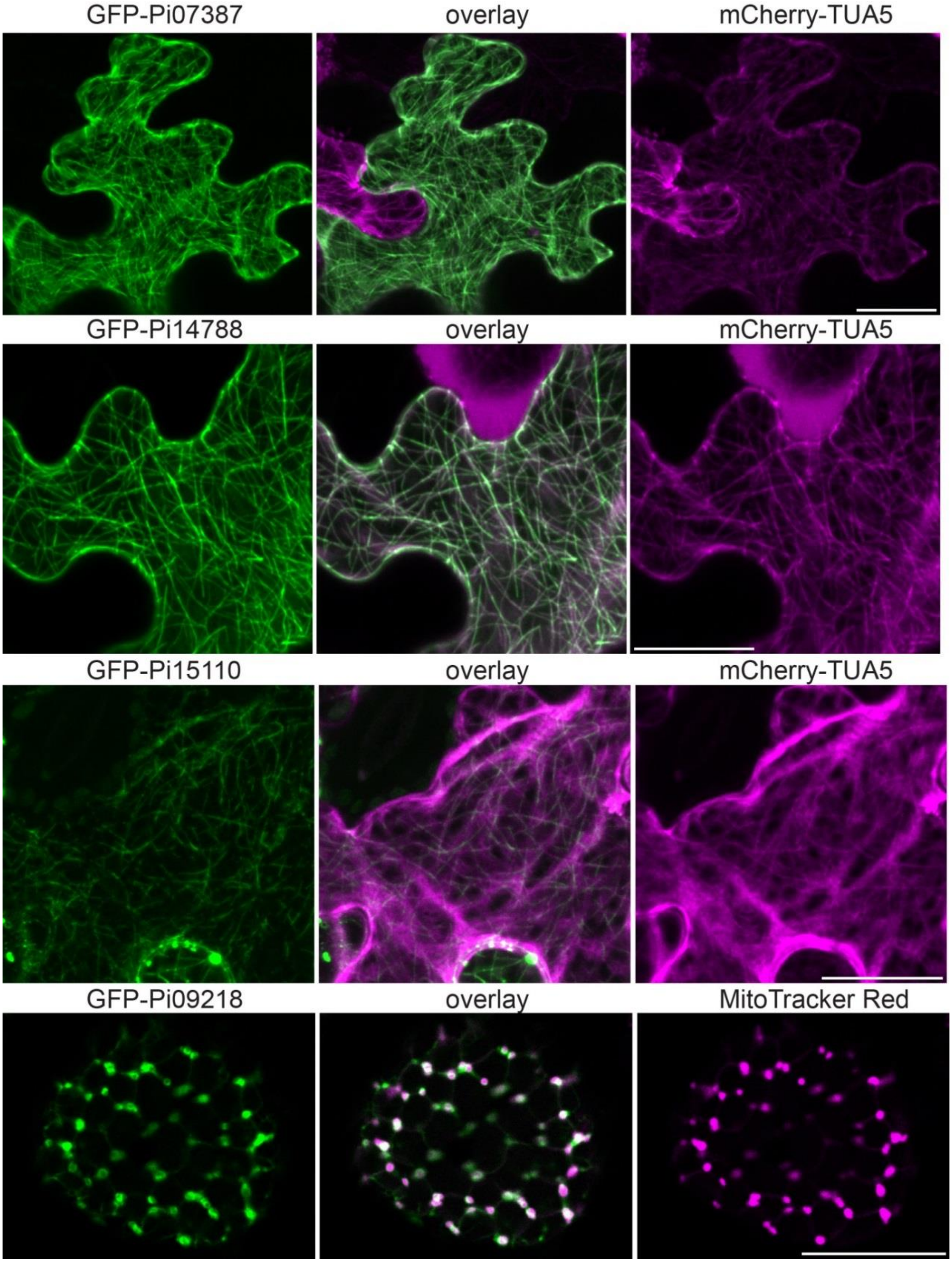

Figure S5 Effector fusions associating with microtubules and mitochondria.

Representative confocal projection images of cells expressing three GFP-effector fusions that show association with the microtubule cytoskeleton, Pi07387, Pi14788 and Pi15110, and one that associates with the mitochondria. Association with the microtubules is demonstrated by co-expression of the mCherry-tubulin 5 (TUA5) fusion. The mitochondria are stained with MitoTracker Red. Scale bars represent 20 µm.

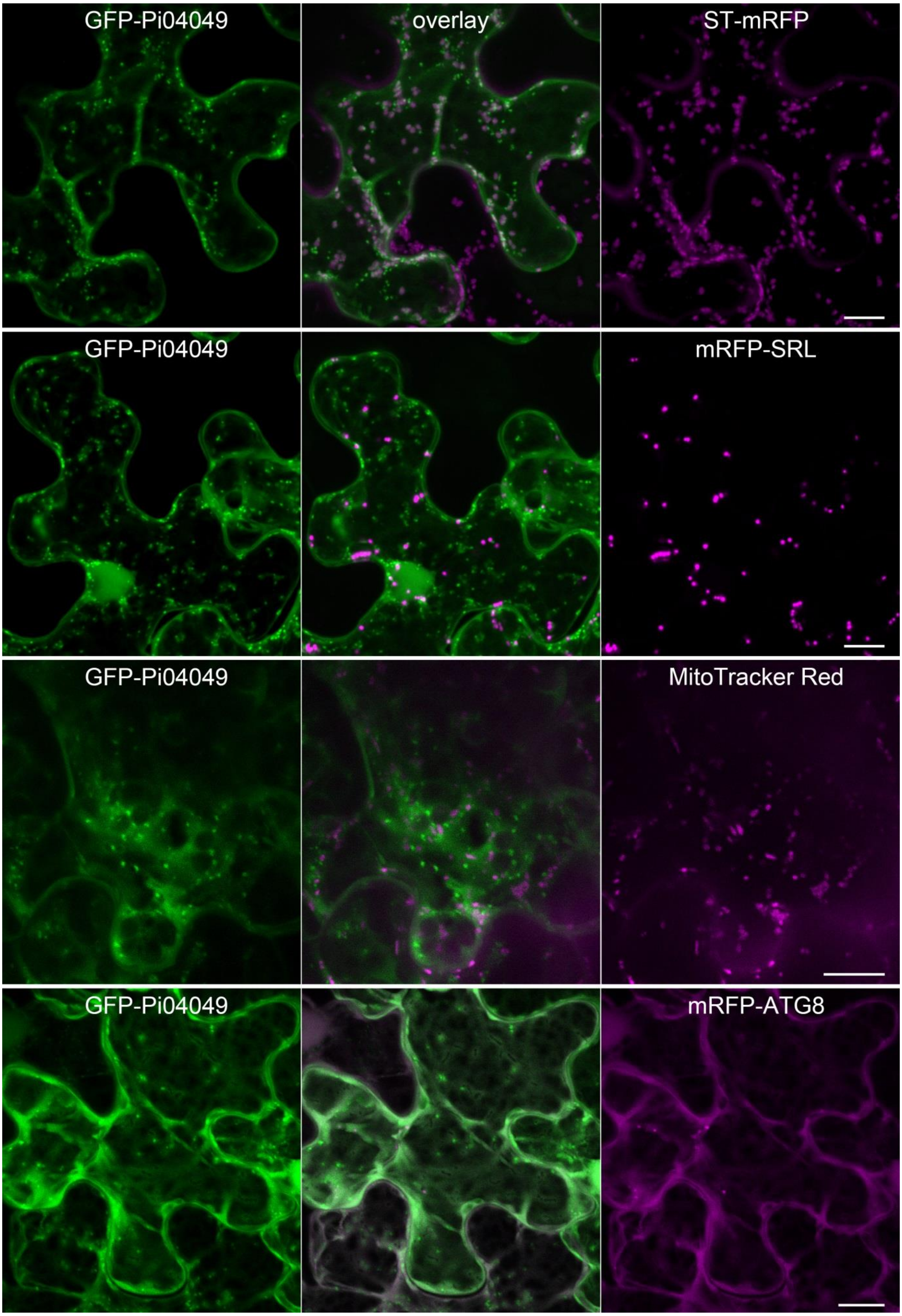
GFP-Pi04049
overlay
ST-mRFP
GFP-Pi04049
mRFP-SRL
GFP-Pi04049
MitoTracker Red
GFP-Pi04049
mRFP-ATG8

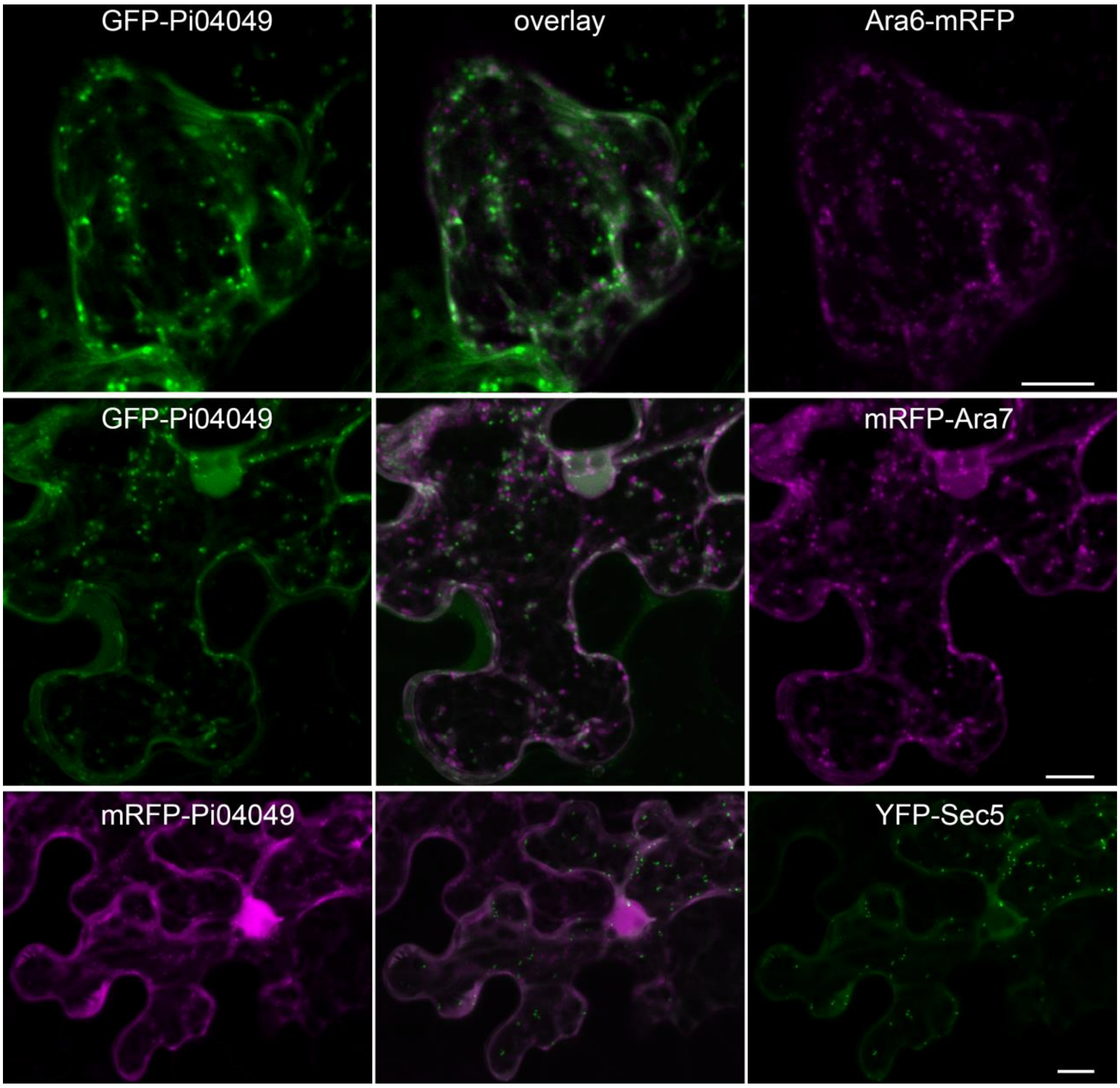

Figure S6 Attempts to identify the GFP-Pi04049-labelled bodies

GFP-04049 or mRFP-04049 was co-expressed with a range of subcellular body markers: ST-mRFP for Golgi, mRFP-SRL for peroxisomes, MitoTracker Red for mitochondria, mRFP-ATG8 for autophagosomes, Ara6-mRFP and mRFP-Ara7 for endosomes, and exocyst component StSec5 fused to YFP as indicated. None of these markers co-localised with the subcellular bodies labelled by the effector fusion. Scale bars represent 10 µm.

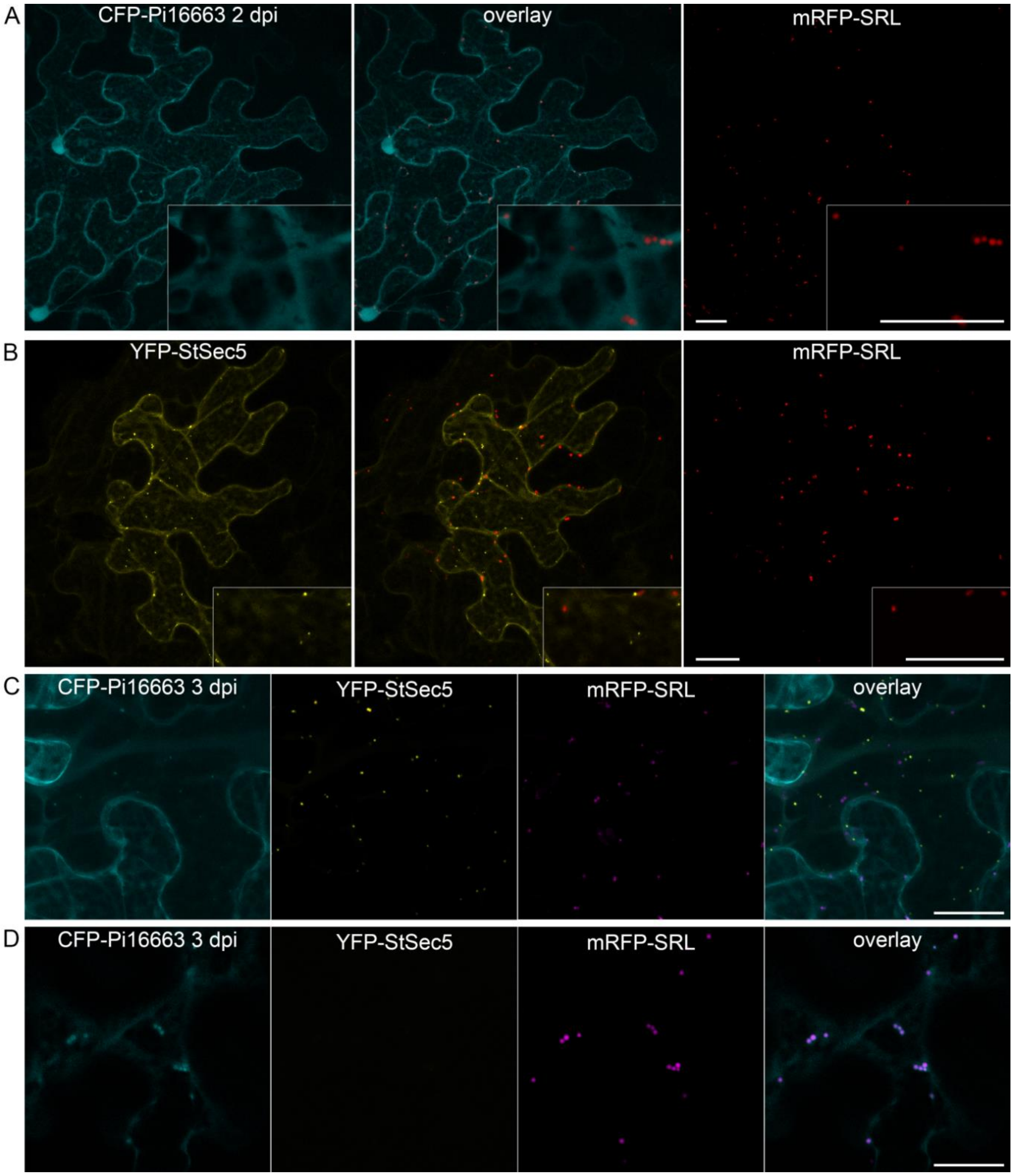

Figure S7 Pi16663 co-localisations

Confocal projection image of CFP-Pi16663 co-expressed with the peroxisome marker mRFP-SRL demonstrates that at 2 dpi the effector fusion does not associate with peroxisomes (A). Co-expression of the YFP-StSec5 fusion with the peroxisome marker demonstrates that the YFP-StSec5 labelled bodies do not associate with peroxisomes (B). Inset images in (A) and (B) are at higher magnification. Co-expression of YFP-StSec5 with the effector fusion and the peroxisome marker at 3 dpi indicates that in cells with readily detectable levels of the YFP-StSec5 fusion the effector fusion co-localises with the YFP-StSec5 bodies (C) whereas in cells in the same infiltration area that do not have detectable YFP the effector fusion is located at peroxisomes (D). Scale bars represent 20 µm.

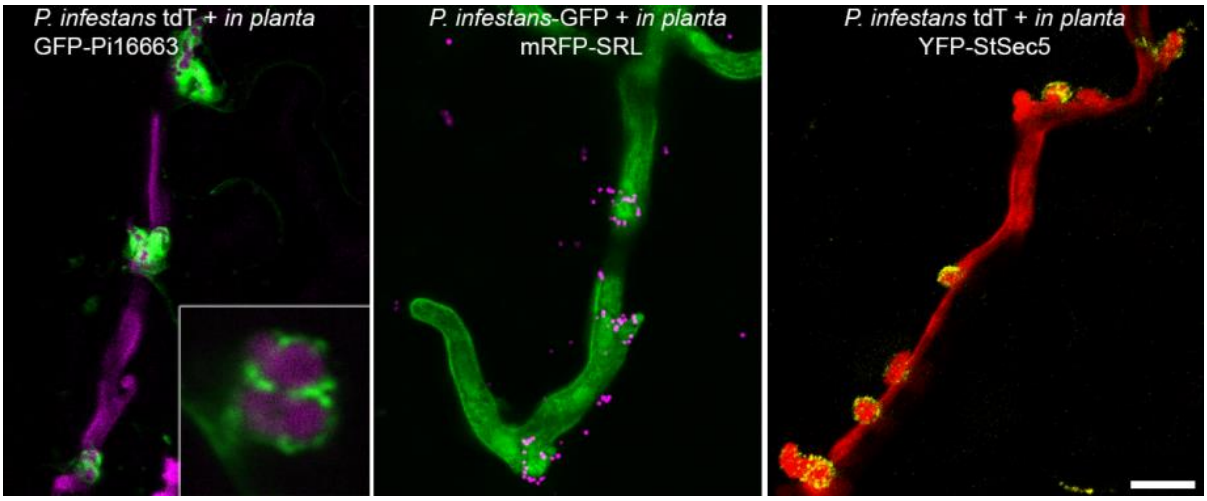

Figure S8 Accumulation of Pi16663 and associated subcellular bodies at haustoria

Confocal projection images of transiently expressed GFP-Pi16663, mRFP-SRL, and YFP-Sec5 around haustoria in leaves co-infected with *P. infestans* expressing tdTomato or GFP as indicated. Inset image is of the GFP-Pi16663 around haustoria at higher magnification. Scale bar represents 10 μm.

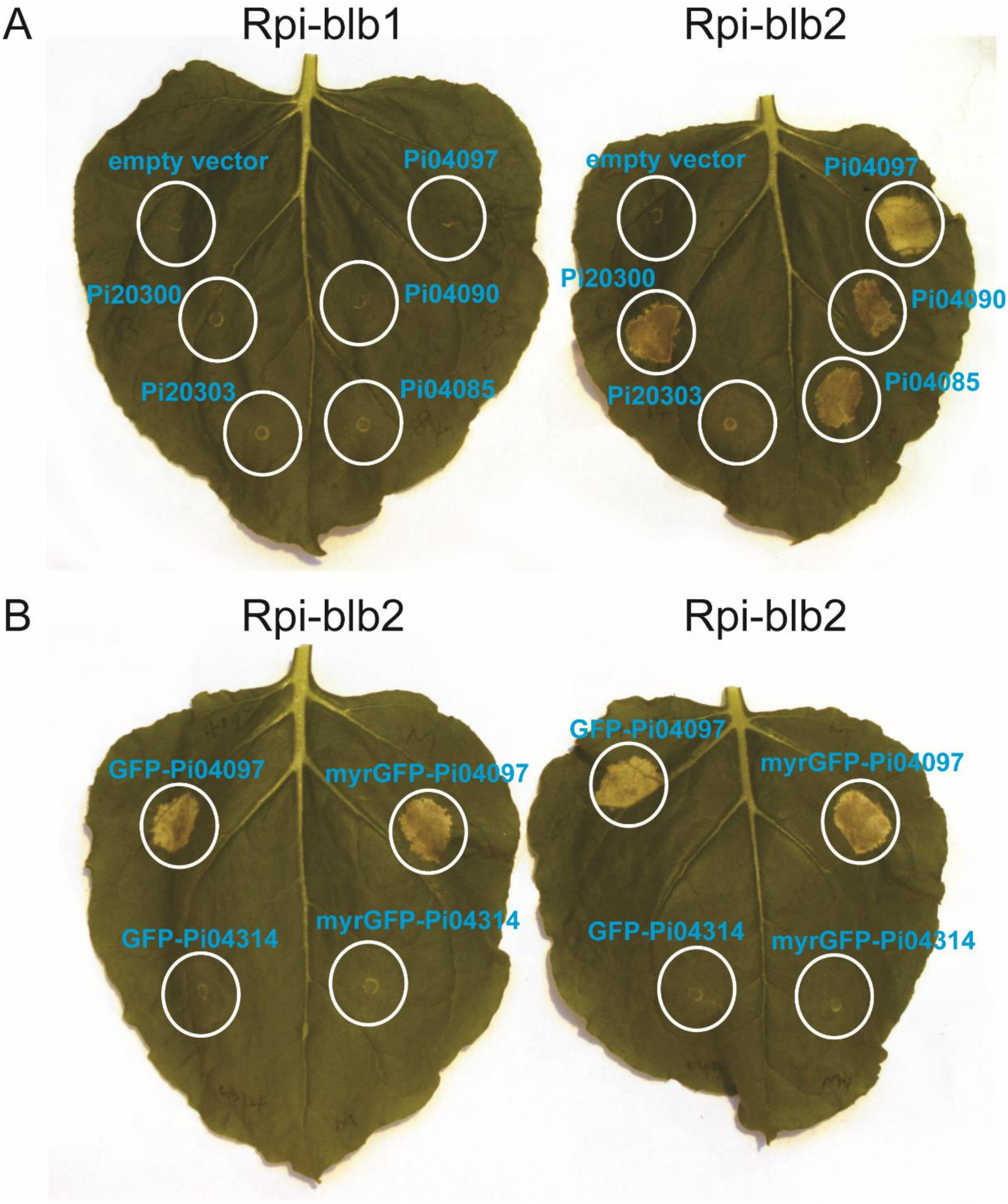

Figure S9 Mis-targeted Avrblb2 family member, Pi04097, is still recognised by Rpi-blb2

Tissue death responses were observed for Avrblb2 family members: Pi04097, 04090, 04085 and 20300 in transgenic *N. benthamiana* expressing Rpi-blb2 but not in plants expressing Rpi-blb1 (A). The regions infiltrated with the controls; an empty vector and an unrecognised family member, Pi20303, did not show cell death responses. The GFP tagged form of Pi04097 also showed a cell death response in the presence of Rpi-blb2 whereas a control GFP-tagged nuclear effector, Pi04314, did not (B). The mis-targeted, form of the Pi04097 fusion, myrGFP-Pi04097 still showed a cell death response with Rpi-blb2. The control myrGFP-Pi04314 did not.

A

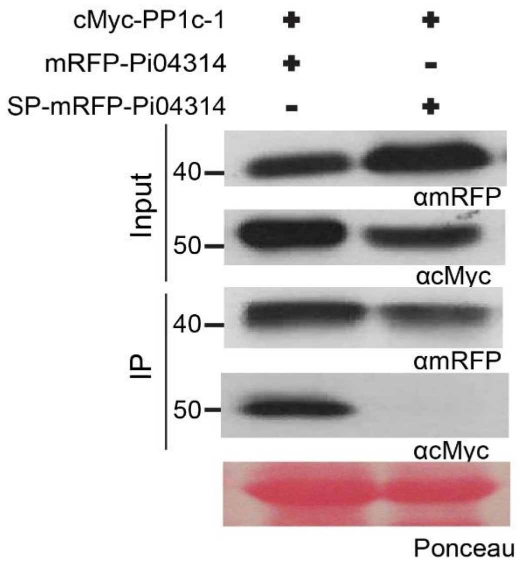

B

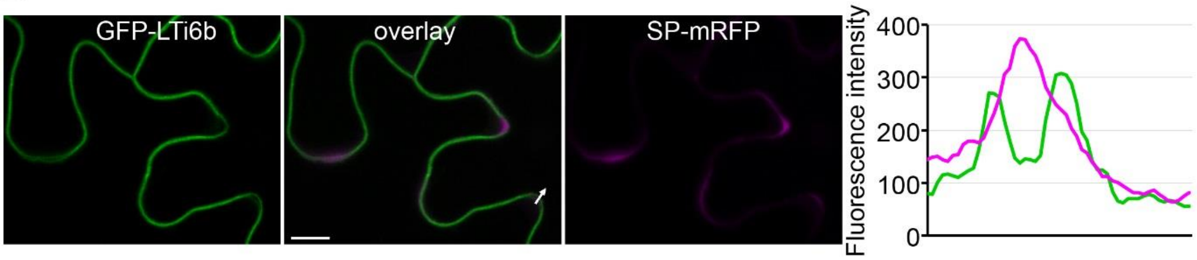

Fig.S10 mRFP- but not SP-mRFP-tagged Pi04314 interacts with its target PP1c-1. Immunoprecipitation (IP) of protein extracts from agroinfiltrated leaves using mRFP–Trap (A). mRFP-Pi04314 expressed within the cell immunoprecipitated PP1c but SP-mRFP-Pi04314 did not. Constructs expressed in the leaves are indicated by +. Protein size markers are indicated in kDa, and protein loading is indicated by Ponceau stain. αcMyc and αmRFP primary antibodies were used as indicated. Example single optical section confocal image of secreted mRFP (SP-mRFP) expressed in transgenic *N. benthamiana* expressing the GFP-LTi6b PM tag for comparison with SP-mRFP effector fusions (B). The arrow indicates the path used to generate the fluorescence intensity profile shown to the right of the image, the x axis represents the length of the arrow. Scale bar indicates 10 μm.

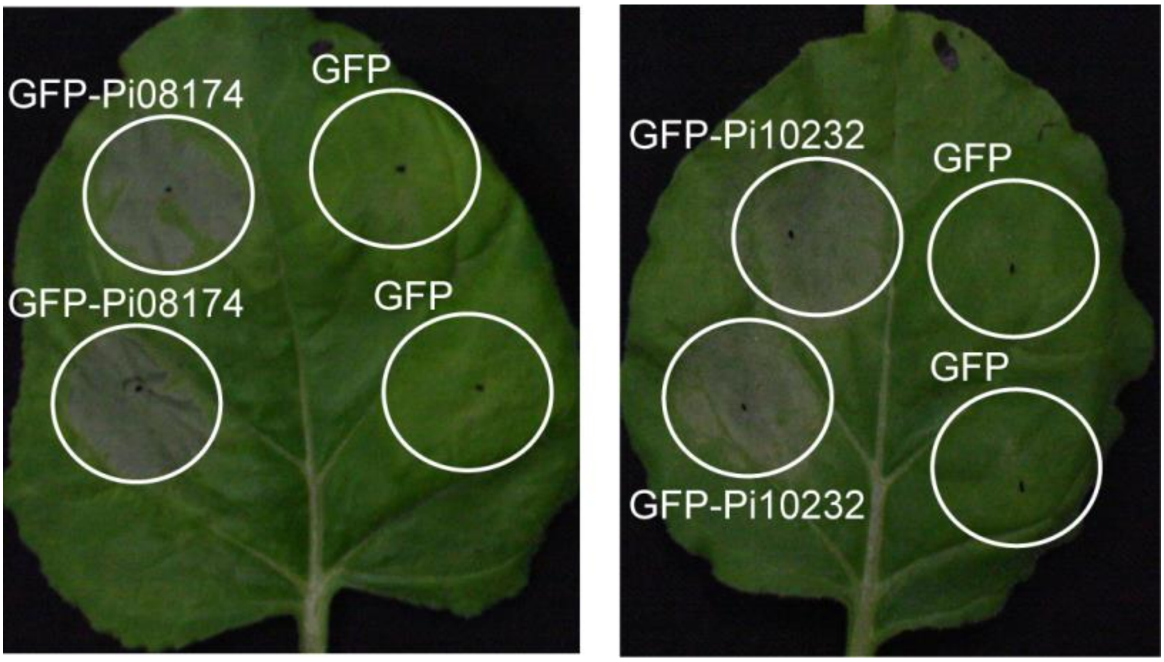

Fig.S11 Effectors that trigger cell death symptoms in *N. benthamiana*

Tissue death responses triggered in *N. benthamiana* by transient expression of GFP fusions of two *P. infestans* effectors on the left side of each leaf and a GFP control on the right side of each leaf as indicated. Leaves were photographed 5 d after infiltration. White circles highlight infiltrated areas.

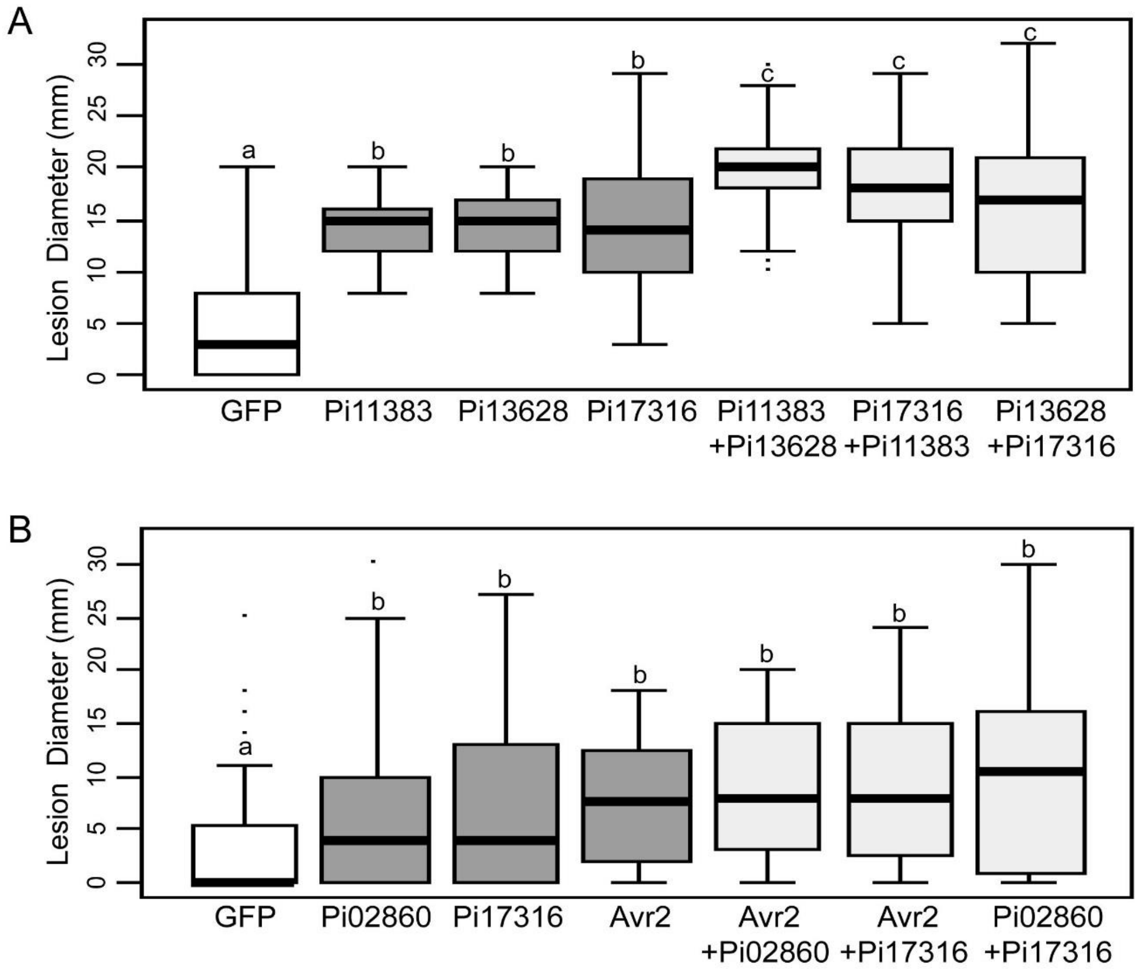

Fig. S12 Effector combination co-expression can provide an additive enhancement of colonisation

Co-expression of effectors that suppress distinct plant immune pathways can provide an additive enhancement of leaf colonisation by *P. infestans,* compared to expression of those effectors alone (A), whereas co-expression of effectors that suppress the same immune pathway does not enhance colonisation beyond that provided by expression of the effectors alone (B). Letters a, b, c denote statistically significant differences ($p<0.05$, ANOVA), where 'a' represents colonisation with GFP control. Data combined from three biological reps ($n = 72$ per construct).
